# Identification of ferroptosis‐related genes in type 2 diabetes mellitus based on machine learning

**DOI:** 10.1002/iid3.1036

**Published:** 2023-10-11

**Authors:** Sen Wang, Yongpan Lu, Tingting Chi, Yixin Zhang, Yuli Zhao, Huimin Guo, Li Feng

**Affiliations:** ^1^ Department of Medical Ultrasound, Shandong Medicine and Health Key Laboratory of Abdominal Medical Imaging, The First Affiliated Hospital of Shandong First Medical University & Shandong Provincial Qian Foshan Hospital Shandong First Medical University Jinan Shandong China; ^2^ Department of Plastic Surgery, The First Clinical Medical College, Shandong University of Traditional Chinese Medicine The First Affiliated Hospital of Shandong First Medical University & Shandong Provincial Qian Foshan Hospital Jinan Shandong China; ^3^ Department of Acupuncture and Rehabilitation The Affiliated Qingdao Hai Ci Hospital of Qingdao University (West Hospital Area) Qingdao Shandong China

**Keywords:** bioinformatics, diagnostic, ferroptosis, gene expression omnibus, machine learning, type 2 diabetes mellitus

## Abstract

**Background:**

Type 2 diabetes mellitus (T2DM), which has a high incidence and several harmful consequences, poses a severe danger to human health. Research on the function of ferroptosis in T2DM is increasing. This study uses bioinformatics techniques identify new diagnostic T2DM biomarkers associated with ferroptosis.

**Methods:**

To identify ferroptosis‐related genes (FRGs) that are differentially expressed between T2DM patients and healthy individuals, we first obtained T2DM sequencing data and FRGs from the Gene Expression Omnibus (GEO) database and FerrDb database. Then, drug‐gene interaction networks and competitive endogenous RNA (ceRNA) networks linked to the marker genes were built after marker genes were filtered by two machine learning algorithms (LASSO and SVM‐RFE algorithms). Finally, to confirm the expression of marker genes, the GSE76895 dataset was utilized. The protein and RNA expression of some marker genes in T2DM and nondiabetic tissues was also examined by Western blotting, immunohistochemistry (IHC), immunofluorescence (IF) and quantitative real‐time PCR (qRT‐PCR).

**Results:**

We obtained 58 differentially expressed genes (DEGs) associated with ferroptosis. GO and KEGG enrichment analyses showed that these DEGs were significantly enriched in hypoxia and ferroptosis. Subsequently, eight marker genes (SCD, CD44, HIF1A, BCAT2, MTF1, HILPDA, NR1D2, and MYCN) were screened by LASSO and SVM‐RFE machine learning algorithms, and a model was constructed based on these eight genes. This model also has high diagnostic power. In addition, based on these eight genes, we obtained 48 drugs and constructed a complex ceRNA network map. Finally, Western blotting, IHC, IF, and qRT‐PCR results of clinical samples further confirmed the results of public databases.

**Conclusions:**

The diagnosis and aetiology of T2DM can be greatly aided by eight FRGs, providing novel therapeutic avenues.

## Graphical abstract

In this study, we identified eight hub genes (SCD, CD44, HIF1A, BCAT2, MTF1, HILPDA, NR1D2, and MYCN) that are closely associated with ferroptosis in T2DM. The three ferroptosis genes (HIF1A, HILPDA, and SCD) are strongly related to T2DM, hypoxia and lipid metabolism, providing new research directions for the development and treatment of T2DM and its complications. Based on these eight genes, we constructed a model with a high ability to diagnose T2DM. We also predicted the drugs corresponding to these eight genes as well as constructed a ceRNA network map. In addition, we verified the protein expression of CD44 and MYCN in T2DM and nondiabetic tissues by Western blotting, Immunohistochemistry staining, Immunofluorescence Staining and quantitative real‐time PCR and the results were statistically significant. The above findings suggest that further studies of ferroptosis may offer new therapeutic goals and biomarkers for patients with T2DM.

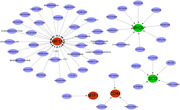

## 1 INTRODUCTION

Diabetes mellitus (DM) is a chronic metabolic disease characterized by high blood glucose levels caused by insulin resistance in peripheral tissues or insufficient pancreatic insulin secretion.^1^ Type 1 diabetes mellitus (T1DM) and type 2 diabetes mellitus (T2DM) are the two primary subtypes of diabetes, with T2DM accounting for nearly 90% of all cases.^2^, ^3^ As living standards improve, the population ages, and the global pandemic of nonalcoholic fatty liver disease (NAFLD) emerges, the prevalence of T2DM will rapidly rise worldwide.^4^, ^5^ According to estimates, the worldwide prevalence of T2DM was 9.3% (463 million) in 2019, and it will be 10.2% (578 million) in 2030 and 10.9% (700 million) in 2045^6^ Additionally, diabetes has a wide range of intricate side effects, including macrovascular conditions such as coronary heart disease, peripheral artery disease, and stroke, as well as microvascular conditions such as diabetic nephropathy, peripheral neuropathy, and retinopathy.^7^, ^8^ Some patients do not know they have T2DM until they start experiencing major side effects. Therefore, finding potential biomarkers and understanding the molecular causes of T2DM is essential for early detection and the avoidance of its consequences.

Ferroptosis was first proposed in 2012 and is a type of iron‐dependent controlled cell death that is accompanied by an aberrant buildup of lipid reactive oxygen species (L‐ROS)^9^, ^10^ Most of the early research on ferroptosis focused on malignancies.^11^ According to an increasing body of research, ferroptosis also plays a significant role in the onset of nonneoplastic disorders, such as Parkinson's disease,^12^ Alzheimer's disease,^13^ pulmonary fibrosis,^14^ and brain damage.^14^ Additionally, elevated ferritin levels have been seen in people with T2DM and gestational diabetes, indicating a link between excess iron storage and the onset of T2DM.^15^, ^16^ Erastin, a ferroptosis inducer, influences the development and operation of human pancreatic islet‐like cell clusters by specifically inhibiting the Xc‐cystine/glutamate antiporter necessary for GSH biosynthesis.^17^, ^18^ In vitro erastin treatment of human islet cells resulted in considerably lower glucose‐stimulated insulin secretion (GSIS) capability. However, GSIS damage was prevented by pretreatment with a ferroptosis inhibitor, such as Fer‐1 or DFO^19^ According to recent research, various T2DM medications in the market can prevent ferroptosis. For instance, ACSL4 is a crucial element of ferroptosis, and rosiglitazone is the most potent ACSL4 inhibitor.^20^, ^21^ However, numerous genes involved in ferroptosis in T2DM have not yet been discovered, necessitating more research on these genes.

The mechanism of ferroptosis in the pathogenic phase of T2DM and the associated consequences are the major focus of current studies on ferroptosis and T2DM. This study sought to add to previous research and establish ferroptosis as a therapeutic target for T2DM by examining the relationship between genes relevant to ferroptosis and T2DM. Cytoscape software was also used to create a drug‐gene interaction map. Then, to investigate the possible regulatory effects of microRNAs (miRNAs) and long noncoding RNAs (lncRNAs) on ferroptosis‐related marker genes in T2DM, we built a competitive endogenous RNA (ceRNA) regulatory network of the marker genes. Our findings offer a novel viewpoint for the clinical diagnosis and management of T2DM and may be useful in clarifying the possible contribution of the ferroptosis process to the pathogenesis of T2DM.

## 2 MATERIALS AND METHODS

### 2.1 Data acquisition

The gene expression information of T2DM and normal tissue samples used in this analysis was obtained from the Gene Expression Omnibus (GEO) database, which is accessible at https://www.ncbi.nlm.nih.gov/geo/. The RNA sequences of 68 T2DM and 62 normal samples are included in the GSE78721 dataset. This dataset served as a training set for the main body of this research study. The expression of the marker genes was validated using the GSE76895 dataset, which included samples from 32 normal individuals and 36 T2DM patients. Additionally, FerrDb (http://www.zhounan.org/ferrdb/) was utilized to obtain the ferroptosis‐related genes (FRGs) (*n* = 358) used in this investigation. These FRGs included the three categories of driver, suppression, and marker FRGs.

### 2.2 Screening ferroptosis‐related differentially expressed genes (DEGs)

We examined DEGs between T2DM samples and normal samples using the limma package in R software. Significant genes were those with *p* < .05. A heatmap was then used to illustrate the overlap of DEGs and FRGs.

### 2.3 Ferroptosis‐related DEGs: Functional enrichment analysis

Gene Ontology (GO) and Kyoto Encyclopedia of Genes and Genomes (KEGG) enrichment analyses of ferroptosis‐related DEGs were performed in R using the clusterProfiler package.^22^ The GO analysis covered three categories: biological processes (BPs), cellular components (CCs), and molecular functions (MFs). These categories are useful in exploring biological functions.^23^ KEGG analysis was utilized to investigate probable biological activities, illnesses, substances, and drugs.^24^

### 2.4 Identification of optimal diagnostic gene biomarkers for T2DM

The glmnet package was used to minimize the data dimensions by employing the least absolute shrinkage and selection operator (LASSO) method.^1^, ^25^ The ferroptosis‐related DEGs found in the T2DM and normal samples were then cross‐validated using the LASSO logistic regression approach to look for disease hallmark genes. Simultaneously, a support vector machine‐recursive feature elimination (SVM‐RFE) model was created using SVM software, and the average misjudgment rates of their 10‐fold cross‐validations were compared.^26^ Finally, the outputs of the LASSO and SVM‐RFE algorithms were intersected to select the best T2DM biomarkers, which were depicted by a Venn diagram. The logistic regression models based on these genes were created using the glmnet package of the R programming language. The diagnostic effectiveness of the logistic regression models was assessed by computing receiver operating characteristic (ROC) curves and calculation of the area under the curve (AUC). ROC curves were also used to assess the ability of a gene to distinguish between samples with and without T2DM.

### 2.5 Single‐gene set enrichment analysis (GSEA)

To further explore the related pathways of the eight marker genes, we performed GSEA to identify pathways enriched in T2DM patients, and “c2.cp.kegg.v7.0.symbols.gmt” from the MSigDB database was adopted as the reference dataset. Patients were divided into high‐ and low‐expression groups according to the expression levels of the eight marker genes. Annotated gene sets were used to distinguish subtypes by the identified DEGs. We computed the consistency P value for each gene set, and *p* values less than .05 were considered significantly enriched. Subsequently, significantly enriched gene sets were sorted according to their correlations from top to bottom.

### 2.6 Single‐gene enrichment analysis using gene set variation analysis (GSVA)

The enrichment of transcriptomic gene sets may be determined using the nonparametric, unsupervised GSVA approach. To assess the biological activities of the samples, GSVA first translates gene‐level changes into pathway‐level changes by rating the sets of genes^27^ We used the KEGG pathway set as the background gene set for this analysis. GSVA evaluation of every marker gene was performed. The possible biological function alterations of various samples were assessed at the same time as the GSVA score difference between samples from the high‐ and low‐expression groups of the marker gene were analysed using limma software.

### 2.7 Immune infiltration analysis

Immune cell infiltration was calculated using the bioinformatics method CIBERSORT (https://cibersortx.stanford.edu/), which was used to quantify the relative proportions of the 22 infiltrating immune cell types in the GSE78721 dataset. The total number of all examined immune cell type fractions for each sample was 1. Using violin plots created using the vioplot program, the differences in immune cell infiltration between T2DM patients and control individuals are shown. Furthermore, immune cells and gene expression levels were analysed using Spearman correlation in this study.

### 2.8 Establishment of a nomogram

Using the rms package, marker genes were included to create a nomogram. In addition, we assessed the accuracy of this nomogram using calibration curves, decision curves, and clinical impact curves.

### 2.9 Drug‐gene interaction

Drugs that modulate marker genes were screened using the Drug Gene Interaction Database (DGIdb). This final medicine list only contained medications that were DrugBank‐sourced and authorized by the Food and Drug Administration.

### 2.10 Construction of the ceRNA network

To predict the binding of marker genes to miRNAs, we used three different programs (miRanda, miRDB, and TargetScan) that all regarded marker genes as miRNA target genes. The spongeScan database provided us with the targeting connection between miRNA and lncRNA. Then, using the Cytoscape program, we built the lncRNA‒miRNA‐messenger RNA (mRNA) regulatory network.

### 2.11 Patients and tissue samples

Diabetic ulcers and normal skin tissues were prospectively collected from six patients who were enrolled in Shandong Provincial Hospital of Traditional Chinese Medicine, Jinan, China. Tissue after ulcer debridement surgery in three patients with diabetes and after circumcision surgery in three patients without diabetes. Half of the above tissue was divided into two parts. These parts were rapidly frozen in a 1.5 mL snap cap tube in liquid nitrogen and stored at −80°C for subsequent molecular analysis. This study was conducted in accordance with the Declaration of Helsinki. All experiments were approved by the ethics committee of the Affiliated Hospital of Shandong University of Traditional Chinese Medicine & Shandong Provincial Hospital of Traditional Chinese Medicine (Approval Number: AF/SC‐08/02.0) and were performed in accordance with the guidelines and regulations.

The other half of the above tissue was fixed in 10% paraformaldehyde fixative, embedded in paraffin and sectioned at 3 μm for histochemical analyses, which included haematoxylin eosin (H&E) staining and immunohistochemical (IHC) staining. Deparaffinization, rehydration, antigen retrieval, endogenous peroxidase blocking, and goat serum (#SP‐9001; ZSGB‐BIO) blocking of paraffin sections were performed. Next, the skin wound tissue sections were incubated with primary antibodies against CD44 (60224‐1‐Ig; Proteintech) and MYCN (ab16898; Abcam) at a dilution of 1:200 at 4°C overnight. On the second day, all sections were incubated with biotin‐labeled goat anti‐mouse immunoglobulin G polymer for 15 min at room temperature and incubated with horseradish enzyme‐labeled streptavidin working solution for 15 min at room temperature. Finally, the slides were counterstained with diaminobenzidine (DAB; #ZLI‐9018, ZSGB‐BIO) and haematoxylin (CAS. 517‐28‐2; Beijing Solarbio Science & Technology). For H&E staining, after deparaffinization and rehydration, slides were stained with haematoxylin and eosin (CAS. 17372‐87‐1; Beijing Solarbio Science & Technology) according to the manufacturer's instructions. All the sections were then dehydrated, cleared, and sealed. The images were observed and captured using an Olympus IX73 microscope (Olympus). ImageJ software was used to calculate the positive rate.

### 2.12 Western blot analysis

Total protein was extracted from tissue samples using RIPA lysis buffer (Beyotime Biotechnology) supplemented with Thermo Scientific Halt Protease Inhibitor Cocktail (Thermo Fisher Scientific). Protease inhibitors and phosphatase inhibitors (1:100) were added during protein extraction (MedChemExpress), and a Pierce BCA Protein Analysis Kit (Thermo Fisher Scientific) was used to measure protein concentrations. Protein samples were separated by 10% sodium dodecyl sulfate–polyacrylamide gel electrophoresis and transferred to polyvinylidene fluoride membranes. The membranes were blocked in 5% skim milk and incubated with the respective primary antibodies overnight at 4°C. The samples were incubated with horseradish peroxidase‐conjugated secondary antibodies (1:5000 dilution; Cell Signaling Technology) for 1 h at room temperature, and an iBright FL1500 imaging system (Invitrogen) and Super Signal West Femto Maximum Sensitivity Substrate (Thermo Fisher Scientific, Invitrogen) were used to detect and analyse protein expression levels. Antibody information was provided for anti‐CD44 (60224‐1‐Ig; Proteintech), anti‐MYCN (ab16898; Abcam), and rabbit anti‐glyceraldehyde 3‐phosphate dehydrogenase (GAPDH) (WB: 1/1000; Cell Signaling Technology).

### 2.13 IHC and immunofluorescence (IF) staining

We performed IHC and IF staining of tissues. IHC of tissues was performed as described previously. For IF staining, paraffin sections were dewaxed in water, and the sections were sequentially placed in environmentally friendly dewaxing solution I for 10 min and then in environmentally friendly dewaxing solution II for 10 min. The sections were then washed with anhydrous ethanol I for 5 min, anhydrous ethanol II for 5 min, anhydrous ethanol III for 5 min, and distilled water. Antigen repair was then carried out, and the repair was completed by natural cooling. The slides were placed in phosphate buffered saline (PBS) (pH 7.4) and washed three times with shaking on a decolorization shaker for 5 min each time. The sections were slightly shaken dry and then closed with a histochemical pen by drawing circles around the tissue and adding 3% BSA dropwise for 30 min. The sections were incubated overnight at 4°C in a wet box after adding the prepared primary antibody dropwise. The slides were placed in PBS (pH 7.4) and washed 3 times with shaking on a decolorized shaker for 5 min each time. The slides were washed three times in PBS (pH 7.4) on a decolorization shaker for 5 min each time. The slides were then observed and recorded using a Nikon Eclipse Ti2 confocal microscope (Nikon Instruments [Shanghai] Co., Ltd.). The following primary antibodies were used: anti‐CD44 (60224‐1‐Ig; Proteintech) and anti‐MYCN (ab16898; Abcam).

### 2.14 RNA isolation and quantitative reverse transcription PCR (qRT‐PCR)

Total RNA was extracted using RNAiso Plus (9109; Takara) according to the manufacturer's protocol. Sketch™ RT Master Mix (RR036A; Takara) was used to synthesize complementary DNA from RNA via reverse transcription. Quantitative real‐time PCR (qRT‐PCR) experiments were performed using TB‐Green™ Premix™ II (RR820A; Takara). Primers were designed and synthesized by Servicebio. GAPDH was used as an endogenous reference gene. Relative gene expression was determined using the 2^−ΔΔCT^ method. The sequences of the employed PCR primers are shown below.

GAPDH‐F 5′‐GGAAGCTTGTCATCAATGGAAATC‐3′

GAPDH‐R 5′‐TGATGACCCTTTTGGCTCCC‐3′

MYCN‐F 5′‐CGAAACTCTGACTCGGAGGACA‐3′

MYCN‐R 5′‐TGGTCCCTGAGCGTGAGAAA‐3′

CD44‐F 5′‐TGGGTTCATAGAAGGGCACG‐3′

CD44‐R 5′‐CCTTTCTGGACATAGCGGGTG‐3′

### 2.15 Statistical analysis

R software 4.2.1 was used for the statistical analysis. The link between 58 ferroptosis‐related DEGs was discovered using Pearson correlation analysis. The data for all experiments are shown as the means ± standard deviations (*SD*s) of three biological replicates, and all data analyses were performed using GraphPad Prism version 9.0.0 (GraphPad Software). Statistical analysis between groups was performed using Student's *t* test to determine significance. A statistically significant difference was indicated by a *p* value less than .05.

## 3 RESULTS

### 3.1 Identification of ferroptosis‐related DEGs between T2DM and controls

**Figure 1:**
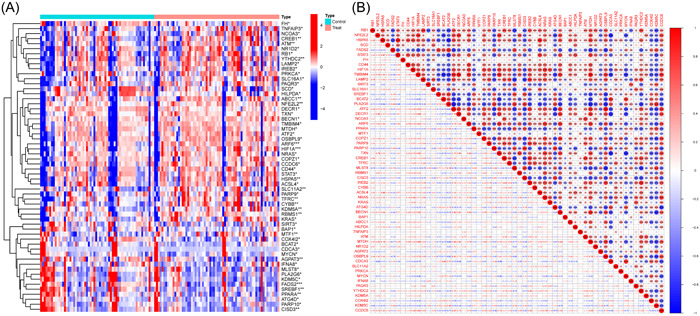
Overview of the differentially expressed ferroptosis genes in T2DM patients and control individuals. (A) Heatmap of 58 ferroptosis‐related DEGs. (B) The correlations among these genes. Most of these genes were strongly correlated with each other. DEG, differentially expressed gene; T2DM, type 2 diabetes mellitus.

In the GSE78721 dataset, 58 FRGs that showed differential expression between T2DM and normal samples were identified; these FRGs included 20 downregulated and 38 upregulated genes (Table 1). The standardized expression of ferroptosis‐related DEGs is displayed in the clustering heatmap of Figure 1A. Figure 1B depicts the interaction between the 58 genes that are associated with ferroptosis in diverse ways. The majority of these genes have a high degree of correlation with one another.

**Table 1:** A total of 58 of 358 FRGs were differentially expressed between T2DM and normal samples, including 38 upregulated and 20 downregulated genes.

Gene	conMean	treatMean	*p* value	Type	Expressing trend
SCD	8.86126	8.27791	.04380	Suppressor	Down
FADS2	6.27324	5.97662	.00036	Driver/Suppressor	Down
FH	7.06160	6.97036	.03202	Suppressor	Down
SIRT3	6.07116	5.94843	.02813	Driver/Suppressor	Down
SREBF1	5.96190	5.65052	.00494	Suppressor	Down
BCAT2	6.45109	6.19764	.02932	Suppressor	Down
PLA2G6	6.17256	5.90314	.04999	Suppressor	Down
PPARA	5.23520	5.11298	.00874	Suppressor	Down
PARP10	6.38217	6.15959	.04404	Suppressor	Down
MLST8	6.54991	6.42335	.03762	Suppressor	Down
CISD3	6.73736	6.44017	.00505	Suppressor	Down
ATG4D	5.45395	5.31774	.04189	Driver	Down
BAP1	4.97176	4.87277	.03871	Driver	Down
HILPDA	7.23339	6.94978	.01367	Driver	Down
AGPAT3	5.59793	5.49169	.00232	Driver	Down
CDCA3	4.10118	3.69864	.03393	Driver	Down
MYCN	3.66731	3.55256	.01115	Driver	Down
IFNA8	2.94453	2.82133	.03202	Driver	Down
COX4I2	5.27891	5.05401	.01043	Driver	Down
KDM5C	5.66135	5.50334	.04478	Driver	Down
RB1	3.98345	4.21985	.01223	Suppressor	Up
NFE2L2	8.16360	8.50608	.00917	Marker/Suppressor	Up
HSPA5	6.29517	6.77562	.00949	Suppressor	Up
STAT3	7.02248	7.34535	.04259	Suppressor	Up
CD44	6.37461	6.84407	.02634	Suppressor	Up
HIF1A	5.64147	6.25449	.00025	Driver/Suppressor	Up
TMBIM4	6.83408	7.25011	.02210	Suppressor	Up
LAMP2	5.04266	5.32603	.02130	Suppressor	Up
SLC16A1	4.61172	4.91838	.03146	Suppressor	Up
ATF2	4.54721	4.70500	.04404	Suppressor	Up
DECR1	8.11819	8.16128	.03109	Suppressor	Up
NCOA3	4.24357	4.37932	.03020	Suppressor	Up
ARF6	6.93977	7.28307	.00024	Suppressor	Up
MTF1	5.12764	5.22054	.00936	Suppressor	Up
COPZ1	7.20000	7.40498	.03128	Suppressor	Up
PARP9	5.21182	5.38351	.01487	Suppressor	Up
TXN	8.43841	8.93875	.03002	Suppressor	Up
CREB1	4.88496	5.16243	.00290	Suppressor	Up
RBMS1	7.64992	7.85439	.00266	Suppressor	Up
IREB2	4.70118	4.85748	.04380	Driver	Up
CYBB	5.78170	6.16301	.00394	Driver	Up
ACSL4	3.90814	4.15309	.04918	Driver	Up
NRAS	5.84546	6.04945	.02847	Driver	Up
KRAS	4.94460	5.10415	.02065	Driver	Up
BECN1	7.25380	7.36999	.01658	Driver	Up
ABCC1	4.92585	5.06815	.00382	Driver	Up
TNFAIP3	5.74721	6.21832	.02169	Driver	Up
ATM	4.12005	4.34600	.00975	Driver	Up
MTDH	6.26852	6.54107	.04918	Driver	Up
NR1D2	4.67254	5.01352	.03183	Driver	Up
OSBPL9	6.33416	6.77677	.02572	Driver	Up
SLC11A2	4.30120	4.37890	.00547	Driver	Up
PRKCA	4.30441	4.37779	.04189	Driver	Up
PAQR3	4.14213	4.23476	.03849	Driver	Up
YTHDC2	4.14048	4.43280	.00789	Driver	Up
KDM5A	5.22178	5.39489	.00123	Driver	Up
CCDC6	5.29112	5.51633	.02378	Driver	Up
TFRC	5.88844	6.53113	.00183	Driver/Suppressor/Marker	Up

Abbreviations: FRG, ferroptosis‐related gene; T2DM, type 2 diabetes mellitus.

### 3.2 Enrichment analysis of ferroptosis‐related DEGs

**Figure 2:**
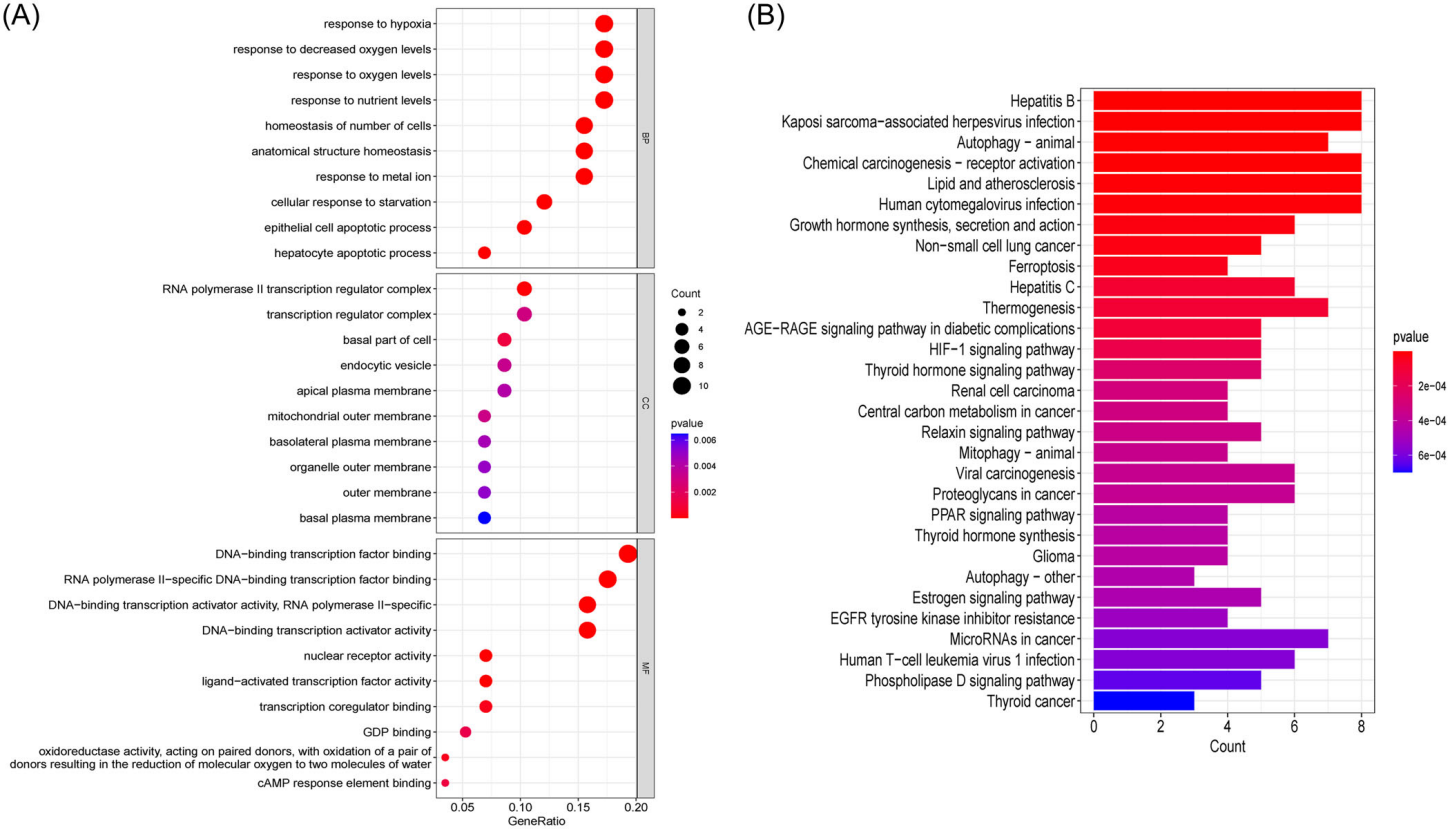
Enrichment analysis of ferroptosis‐related DEGs. (A) Gene Ontology (GO) functional analysis showing enrichment of ferroptosis‐related DEGs. (B) Kyoto Encyclopedia of Genes and Genomes (KEGG) pathway enrichment analysis of ferroptosis‐related DEGs. DEG, differentially expressed gene.

GO and KEGG enrichment analyses were performed to further study the biological activities and pathways of these ferroptosis‐related DEGs, as shown in Figure 2A,B, respectively. The important GO‐BP categories were primarily connected with hypoxia, such as stress hypoxia, reduced oxygen levels, and oxygen levels. The ferroptosis‐related DEGs were highly enriched in the transcription regulator complex and the basal portion of the cell in GO‐CC analysis. The pathways enriched by GO‐MF were mostly related to transcription factors. The top 20 enriched pathways, according to KEGG analysis, were mostly involved in viral hepatitis, growth and thyroid hormone production, autophagy‐related processes, and ferroptosis. Surprisingly, ferroptosis‐related DEGs were clearly enriched in cancer‐related signatures such as non‐small cell lung cancer, renal cell carcinoma, chemical carcinogenesis‐receptor activation, viral carcinogenesis, and central carbon metabolism in cancer. These findings suggest that hypoxia and transcription factors may play a crucial role in the development of T2DM and also provide some novel pathways related the link between T2DM and cancer.

### 3.3 Eight ferroptosis‐related DEGs were identified as diagnostic genes for T2DM

**Figure 3:**
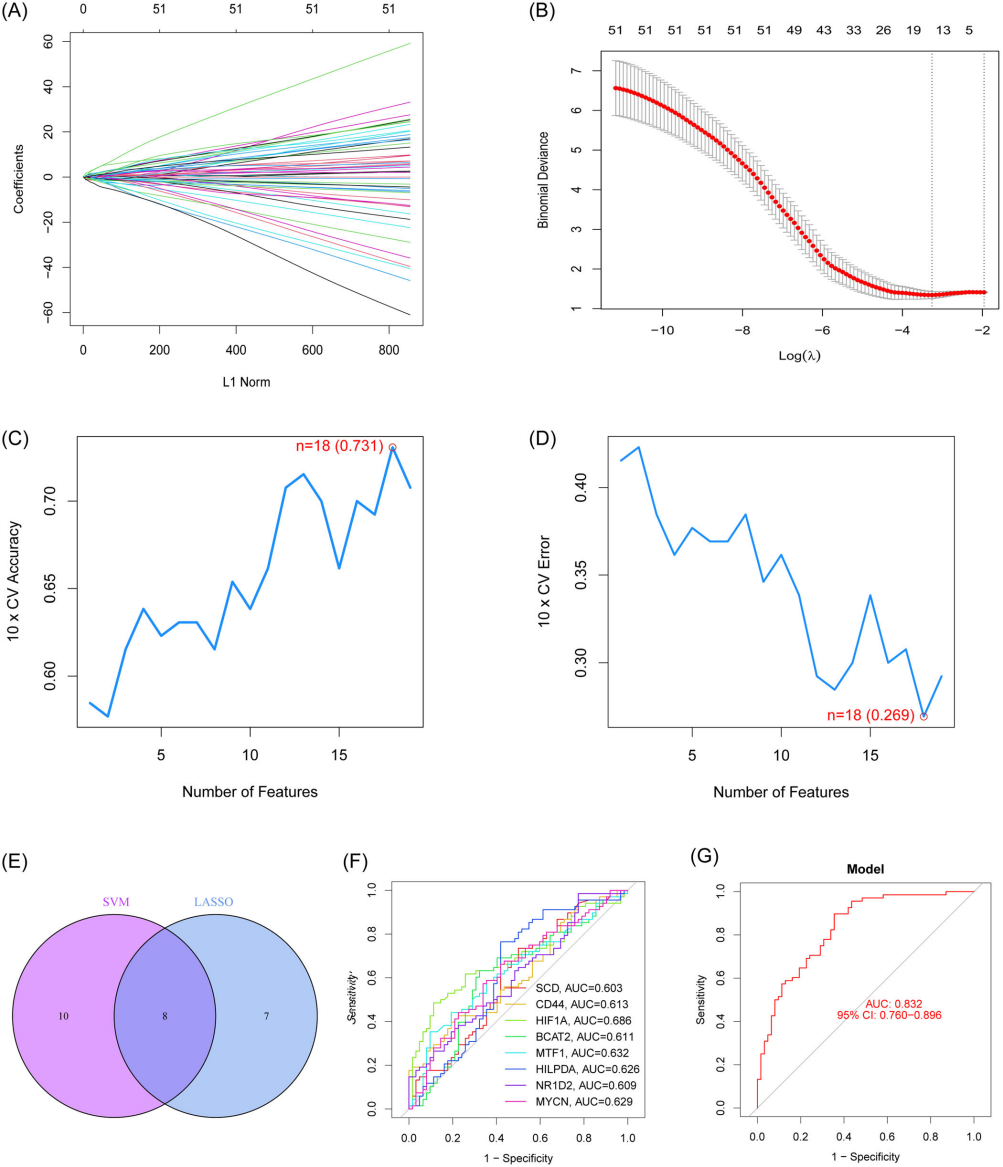
Eight ferroptosis‐related DEGs were identified as diagnostic genes for T2DM. (A and B) Fifteen ferroptosis‐related DEGs obtained using the LASSO algorithm based on the minimum lambda. (C and D) Eighteen ferroptosis‐related DEGs obtained using the SVM‐RFE algorithm (maximum precision = 0.731, minimum RMSE = 0.269). (E) Eight marker genes were obtained from the LASSO and SVM‐RFE algorithms. (F) ROC curves for the 8 marker genes. (G) Logistic regression model to identify the AUC of disease samples. AUC, area under the curve; DEG, differentially expressed gene; RMSE, root‐mean‐square deviation; ROC, receiver operating characteristic curve; T2DM, type 2 diabetes mellitus.

LASSO and SVM‐RFE were used to screen the significant ferroptosis‐related DEGs to distinguish T2DM patients from normal individuals in GSE78721. In the LASSO logistic regression algorithm, we selected the 15 genes at the time of minimum cross‐validation error (Figure 3A,B). The best diagnostic genes for T2DM were ultimately determined to be 18 genes (highest precision = 0.731, minimum root‐mean‐square deviation = 0.269) after using the SVM‐RFE method was used to filter 58 ferroptosis‐related DEGs (Figure 3C,D). The 8 genes (SCD, CD44, HIF1A, BCAT2, MTF1, HILPDA, NR1D2, and MYCN) that overlapped between these two algorithms were selected (Figure 3E).

**Figure 4:**
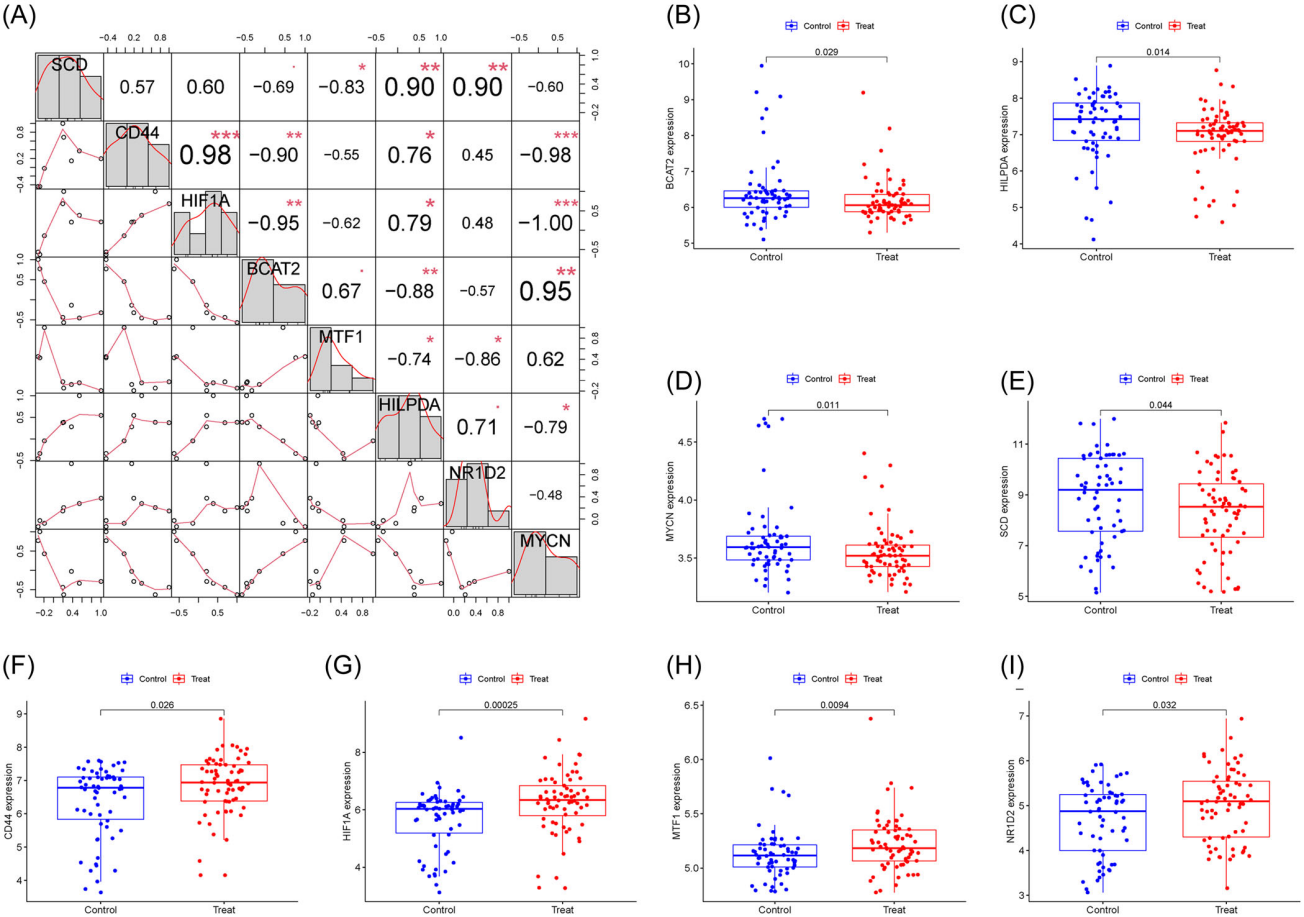
The interaction relationships and expression of the eight marker genes in GSE78721. (A) Interaction of eight marker genes. (B–I) Comparison of the expression of eight marker genes in T2DM and healthy samples. T2DM, type 2 diabetes mellitus.

The AUC for all genes was larger than 0.6 when we drew ROC curves for these eight biomarkers that were identified by both machine learning systems (Figure 3F). We also created a logistic regression model using the R glmnet package based on these eight biomarkers. According to our ROC curve results, the eight marker gene‐based logistic regression model offered more sensitivity and precision than the independent marker genes for discriminating T2DM samples from normal samples, with an AUC of 0.832 (95% confidence interval [CI]: 0.760–0.896) (Figure 3G). Additionally, the interactions and expression levels of the eight marker genes in the GSE78721 dataset are shown in Figure 4.

### 3.4 Various pathways associated with marker genes

**Figure 5:**
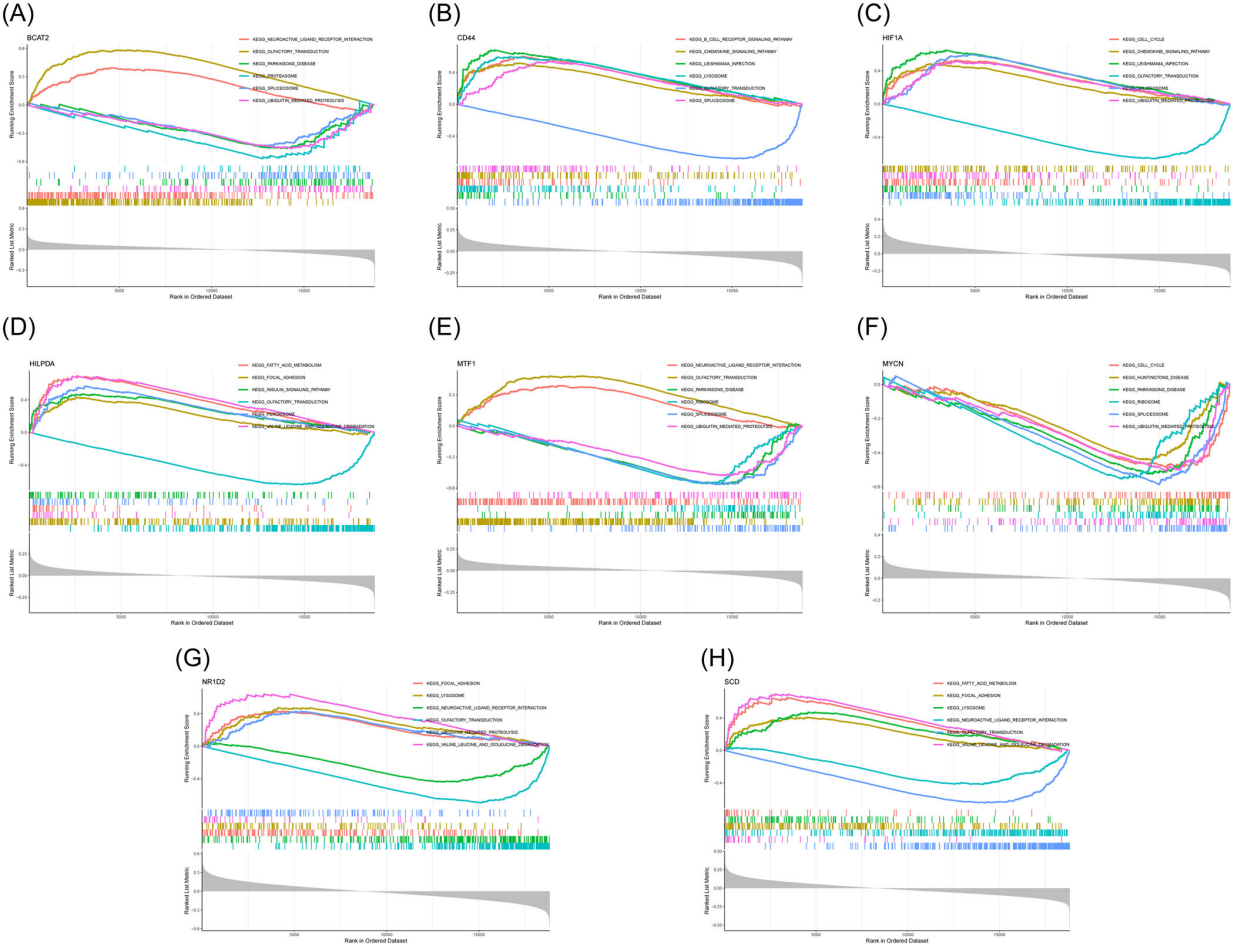
Single gene KEGG‐GSEA of these eight genes. GSEA, gene set enrichment analysis; KEGG, Kyoto Encyclopedia of Genes and Genomes.

We carried out a single‐gene GSEA‐KEGG pathway analysis to further investigate the distinct signaling pathways connected to the marker genes. The first six routes for each marker gene are displayed (Figure 5A–H). After a thorough analysis, we discovered that these eight marker genes were primarily enriched in the pathways related to lysosomes, the cell cycle, ribosomes, peroxisomes, ubiquitin‐mediated proteolysis, and fatty acid metabolism and various disease pathways (including those for Parkinson's disease and Huntington's disease). In addition, we discovered that the marker genes were enriched in the chemokine signaling pathway, B‐cell receptor signaling pathway, olfactory transduction, and neuroactive ligand‒receptor interaction.

### 3.5 GSVA

**Figure 6:**
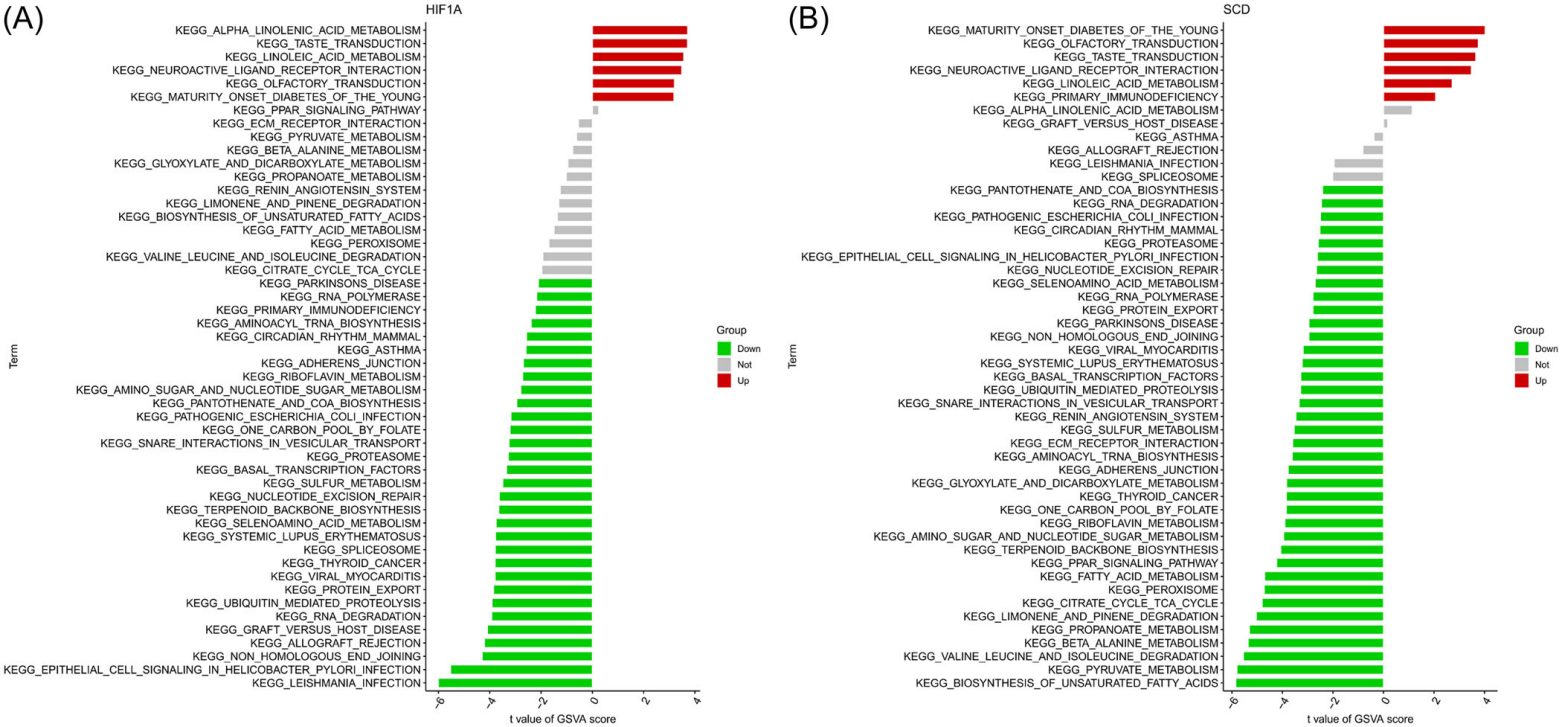
High‐and low‐expression groups based on the expression levels of each marker gene combined with GSVA in HIF1A (A) and SCD (B). GSVA, gene set variation analysis.

Then, we analysed the differentially activated pathways between the high‐ and low‐expression groups according to the expression level of each marker gene in combination with GSVA results (see Figure S1 in the Supporting Information Content, which illustrates the GSVA results of these marker genes). We found that the high expression of CD44 and HIF1A in this disease are highly similar (Figure 6) and probably act through the induction of T2DM by activating linoleic acid metabolism and neuroactive ligand receptor interaction. In addition, they were associated with taste and olfactory transduction. The low expression of HILPDA and SCD in T2DM are also highly similar, and they were mainly related to the synthesis, metabolism and degradation of substances, such as the biosynthesis of unsaturated fatty acids, terpenoid backbone biosynthesis, fatty acid metabolism, beta alanine metabolism, pyruvate metabolism, selenoamino acid metabolism, and valine leucine and isoleucine degradation. In addition, the low expression of SCD and high expression of MTF1 were associated with systemic lupus erythematosus, parkinsons disease, and pathogenic escherichia coliinfection. Low MYCN expression in this disease was only involved in alpha linolenic acid metabolism. High expression of NR1D2 was enriched in olfactory transduction and neuroactive ligand receptor interaction. Notably, BCAT2, CD44 and HIF1A were directly related to the pathway of maturity onset diabetes of the young.

### 3.6 Immune landscape analysis

We investigated immune cell infiltration in T2DM patients and normal control individuals using CIBERSORT. The percentage of immune cells from 62 normal and 68 T2DM samples is shown in Figure 7A. The interactions of the immune cells revealed that resting mast cells and active mast cells had the most pronounced negative correlation, with *r* = .51, while naive CD4 T cells and naive B cells had the most significant positive correlation, with *r* = .83 (Figure 7B). Figure 7C demonstrates that whereas activated dendritic cells (DCs) are more prevalent in T2DM patients, resting DCs are less common in T2DM patients than in normal samples.

Figure 7Immune feature analysis. (A) Bar charts of 22 immune cell proportions in T2DM and control samples. (B) Correlation heatmap depicting correlations among infiltrated immune cells in sepsis. The darker the color is, the stronger the correlation. (C) Differential expression of different types of immune cell markers between T2DM and controls. T2DM, type 2 diabetes mellitus.

**Figure:**
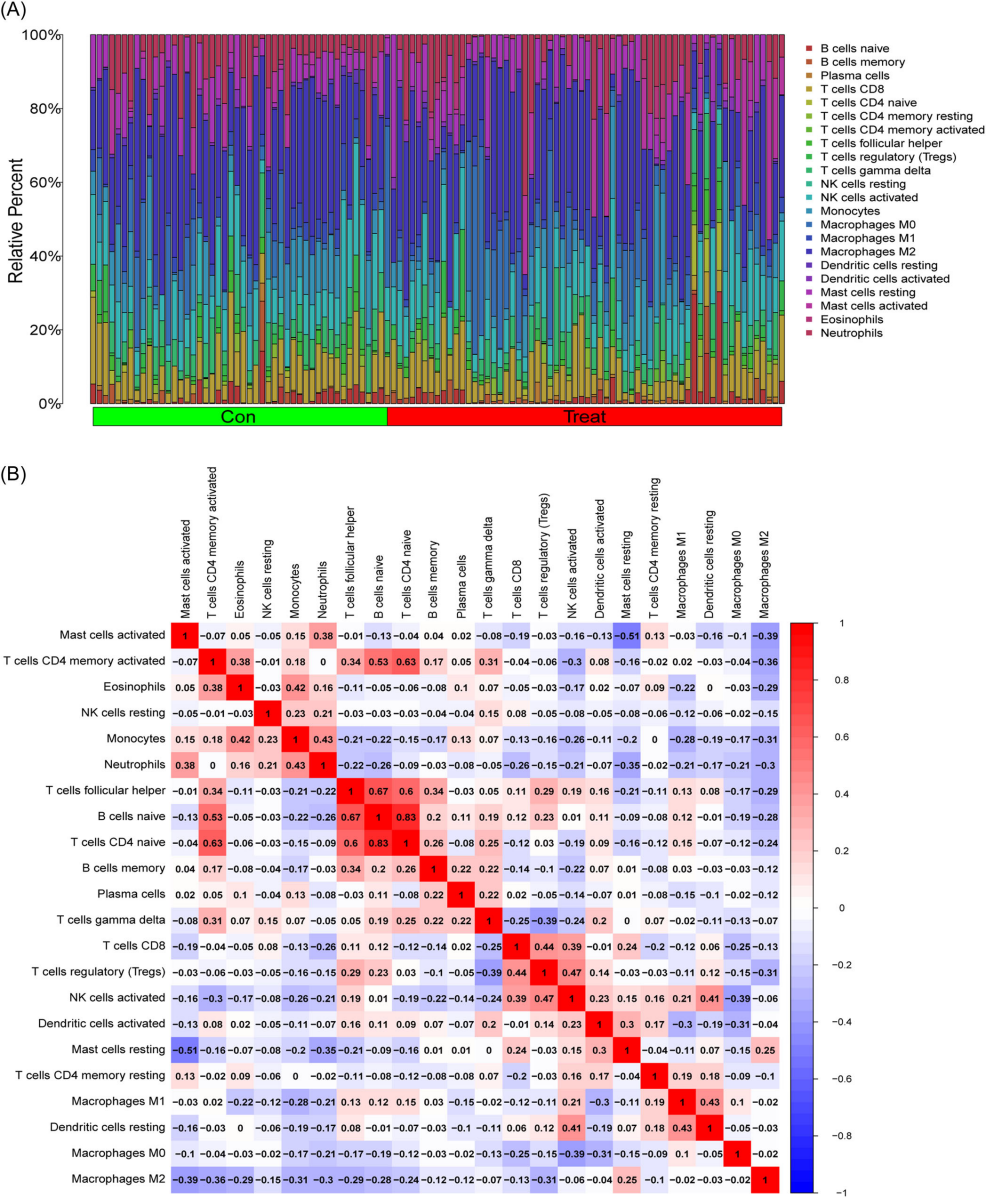

**Figure:**
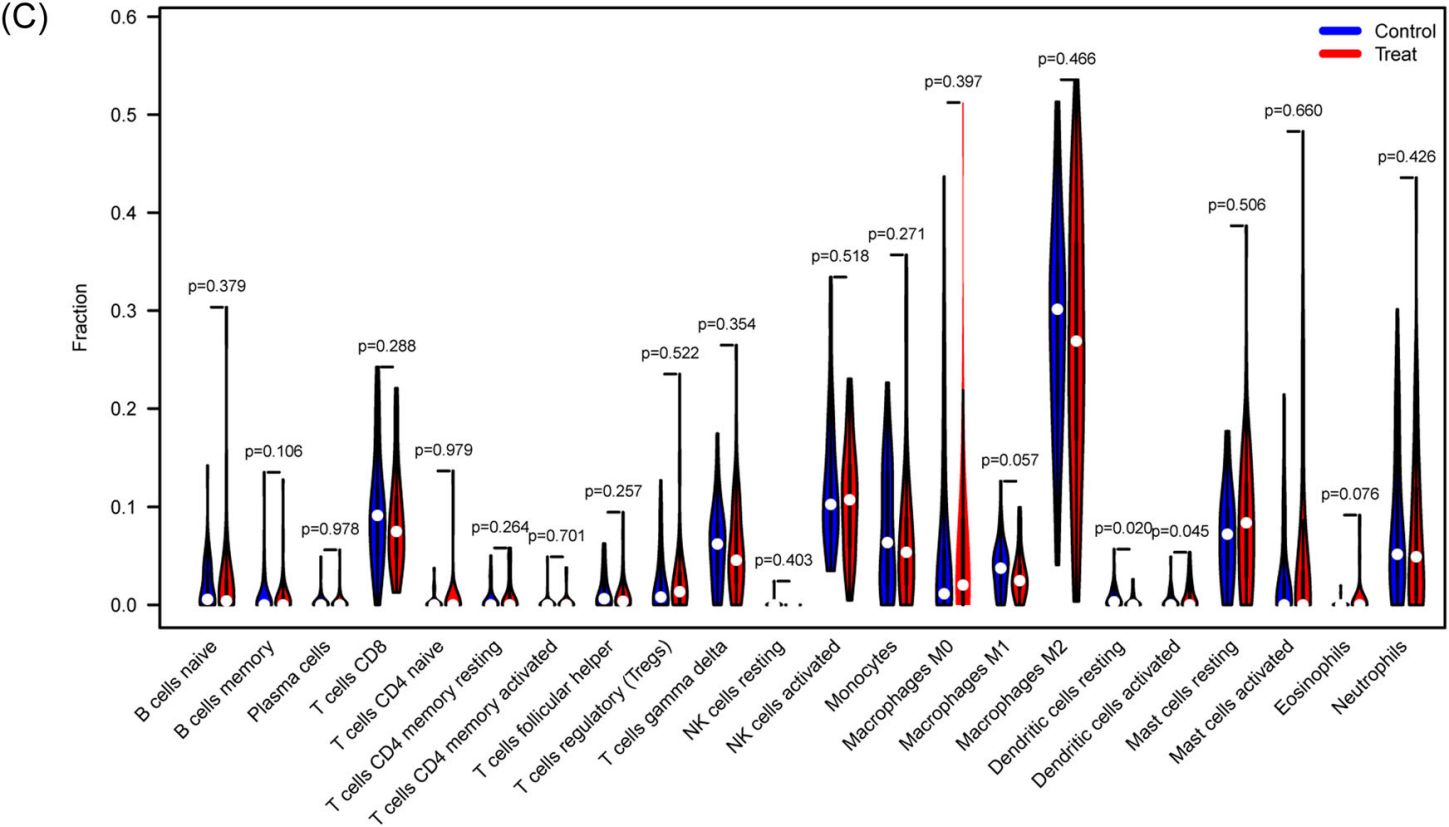

### 3.7 Correlation analysis of the eight biomarkers and infiltrating immune cells

**Figure 8:**
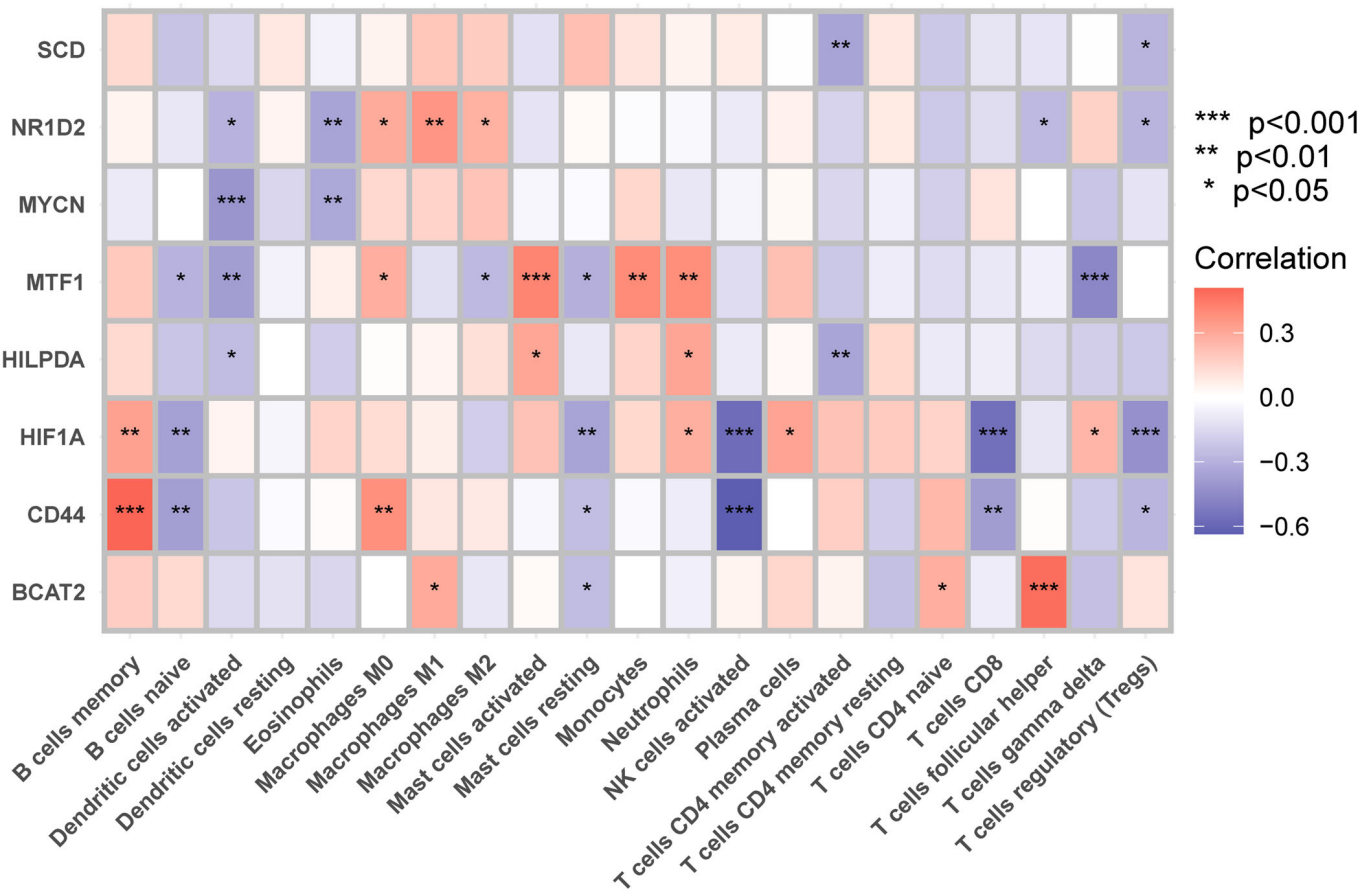
Correlations between the expression of these eight marker genes and immune cells.

As illustrated in Figure 8, the correlation analysis revealed a significant link between these 8 marker genes and immune cells. We discovered that CD44 strongly correlated with the activation of NK cell and B‐cell memory. HIF1A had a substantial negative correlation with both activated NK cells and CD8 T cells. NR1D2, MYCN, MTF1, and HILPDA all had a negative correlation with DC activation. M0 macrophages, activated mast cells, monocytes, and neutrophils all had a positive correlation with MTF1. Of course, marker genes related to neutrophils also include HILPDA and HIF1A. These data imply that these newly discovered marker genes may be linked to alterations in the immune microenvironment in T2DM patients.

### 3.8 Construction and assessment of the nomogram for patients with T2DM

**Figure 9:**
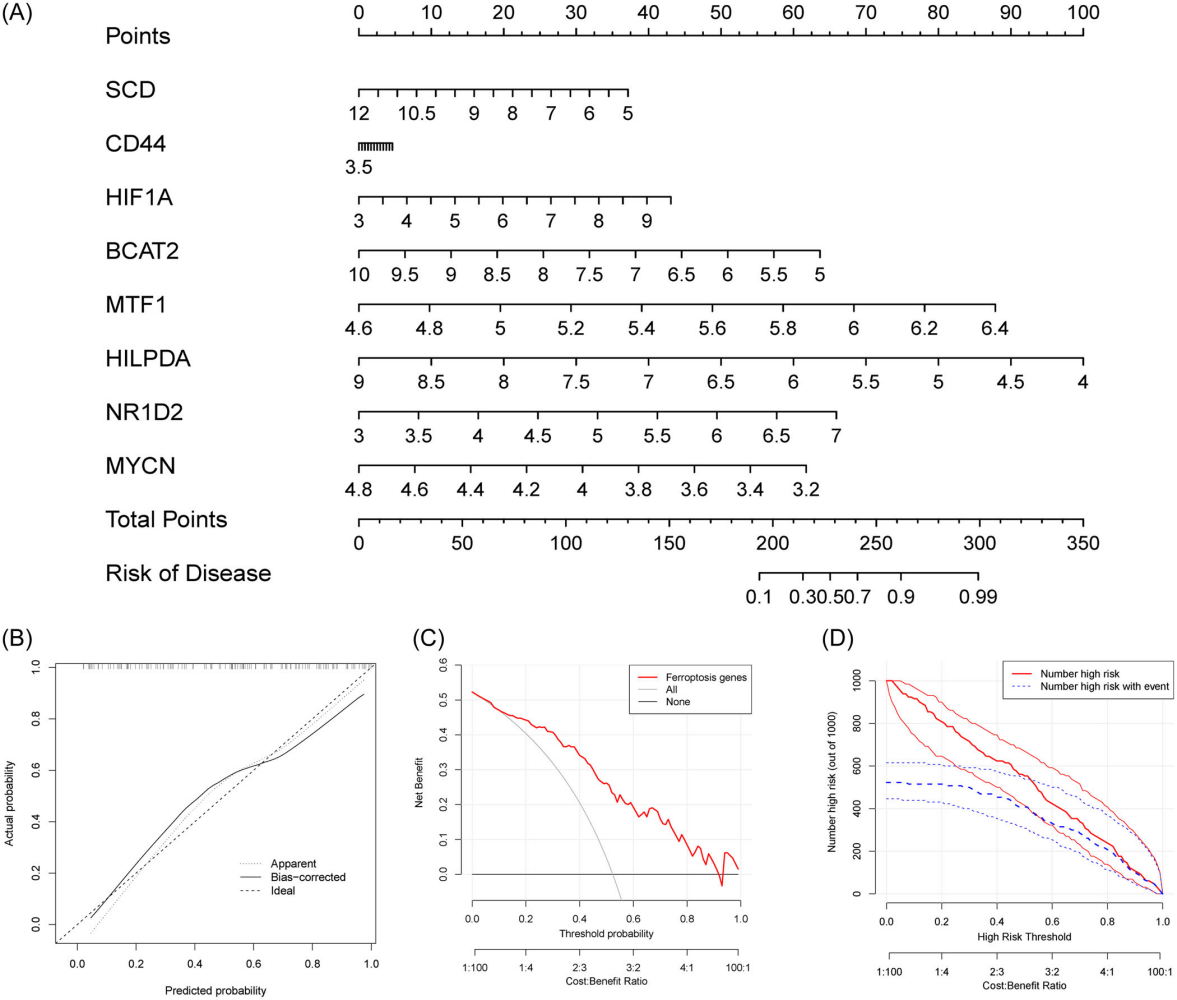
Construction of the nomogram based on the logistic regression model. (A) The nomogram specifically quantified the odds ratio of T2DM based on eight ferroptosis characteristics. (B) The calibration curves of the nomogram. (C) The decision curve of the nomogram. (D) The clinical impact curves of the nomogram. T2DM, type 2 diabetes mellitus.

A nomogram was created as a diagnostic tool for T2DM by including marker genes (Figure 9A). Each marker gene in the nomogram was assigned a score, and the overall score was obtained by adding the scores of all marker genes. Higher overall scores increased the chance of acquiring T2DM, while lower total scores were linked to lower risks of developing T2DM. The high accuracy of the nomogram was shown by the calibration and decision curve (Figure 9B,C). The nomogram also maintained excellent accuracy in identifying high‐risk T2DM patients, as seen in the clinical impact curves (Figure 9D).

### 3.9 Drug‐gene interaction

**Figure 10:**
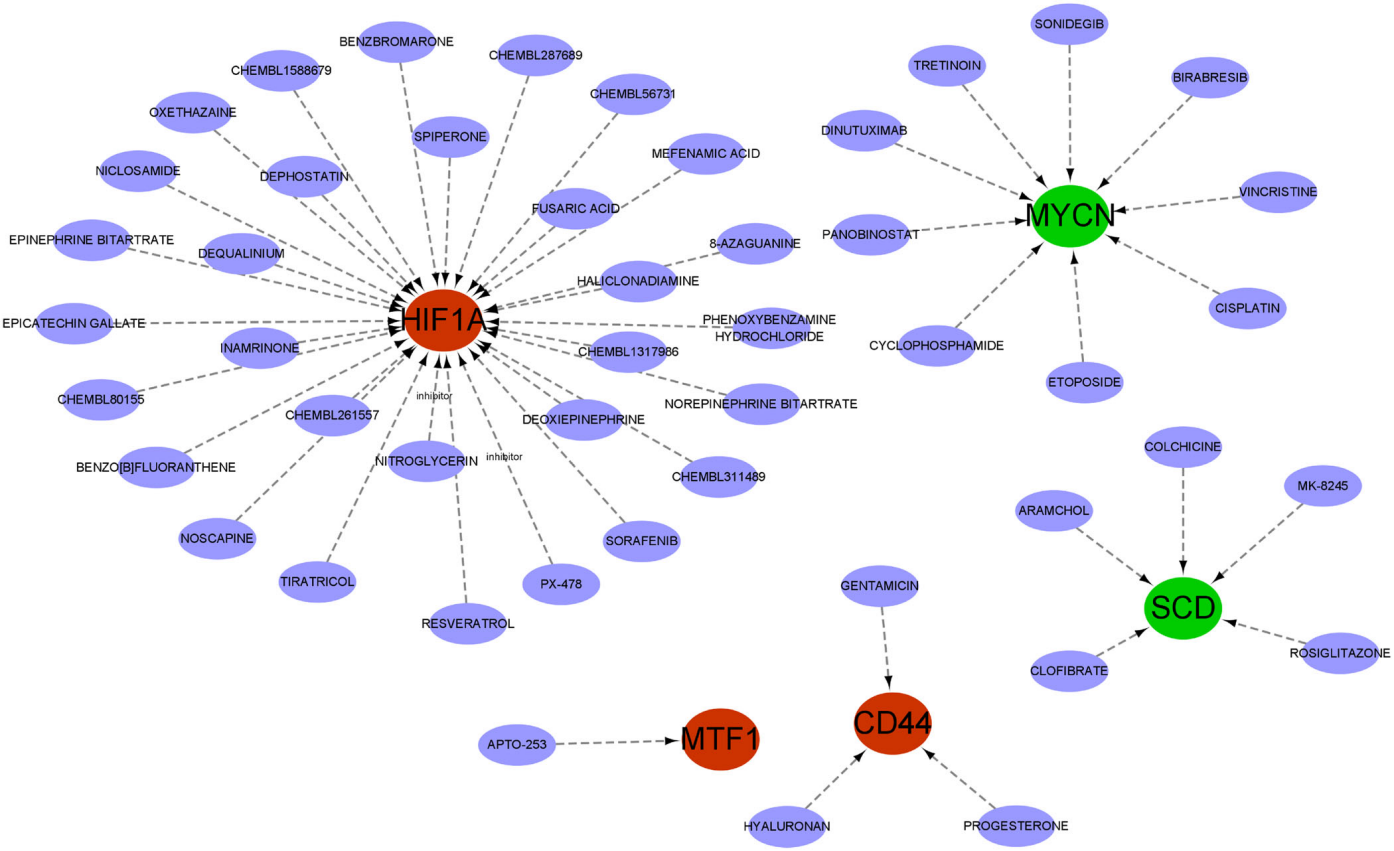
Prediction of marker gene‐targeted drugs. The drugs may target marker genes through the DGIdb database and the interaction relationship between the two.

We searched through the DGIdb database for drugs that might affect the marker genes. The Cytoscape software‐visualized results are displayed in Figure 10. We queried 48 medicines targeting marker genes, including 30 for HIF1A, 9 for MYCN, 5 for SCD, 3 for CD44 and 1 for NTF1. Unfortunately, the medications connected to BCAT2, HILPDA, and NR1D2 were not predicted by us. In addition, we also searched the structural formulae of the above 48 drugs using the DrugBank database. Thirty‐two drug structures in all were found. A total of 16 drug structures were retrieved from 30 HIF1A‐targeted drugs (Figure S2 in Supporting Information Content illustrates the structures of the 32 drugs). Among them, PX‐478 and nitroglycerin are known inhibitors of HIF1A. A total of eight drug structures were retrieved from the 9 targeted drugs of MYCN. The corresponding drug structures were derived for all five targets of SCD and three targets of CD44.

### 3.10 Marker gene‐based ceRNA networks

**Figure 11:**
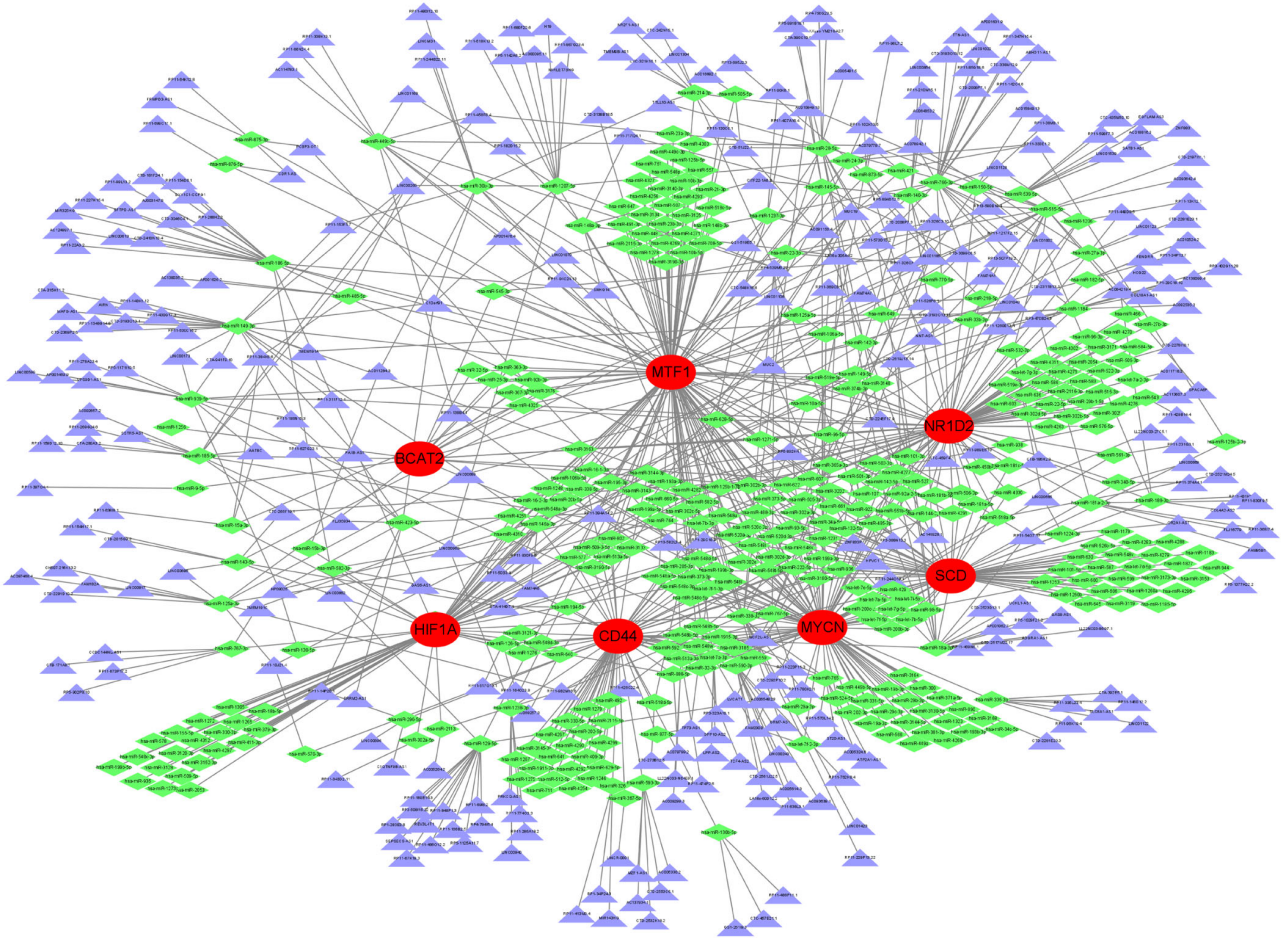
A ceRNA network based on these eight genes. The network includes 671 nodes (7 marker genes, 349 miRNAs and 315 lncRNAs). ceRNA, competitive endogenous RNA; lncRNA, long noncoding RNA; miRNA, microRNA.

We built a ceRNA network based on these eight marker genes to highlight the interactions among lncRNAs, miRNAs, and mRNAs, as shown in Figure 11. The network includes 671 nodes (7 marker genes, 349 miRNAs, and 315 lncRNAs). Eighteen miRNAs were associated with BCAT2, 69 were associated with CD44, 62 were associated with HIF1A, 115 were associated with MTF, 96 were associated with MYCN, 85 were associated with NR1D2, and 70 were associated with SCD. We discovered that 19 lncRNAs could control the expression of SCD and MTF1 through competitive binding to hsa‐miR‐149‐3p, 16 lncRNAs controlled the expression of SCD, MTF1, NR1D2 and HIF1A through competitive binding to hsa‐miR‐149‐3p, 15 lncRNAs controlled the expression of NR1D2, MTF1, and MYCN through competitive binding to hsa‐miR‐515‐5p, 15 lncRNAs controlled the expression of SCD, MTF1, and CD44 through competitive binding to hsa‐miR‐18a‐3p, 8 lncRNAs controlled the expression of MYCN, NR1D2, and CD44 through competitive binding to hsa‐miR‐590‐3p, 14 lncRNAs controlled the expression of MTF1 through competitive binding to hsa‐miR‐129‐5p, and 20 lncRNAs controlled the expression of NR1D2 through competitive binding to hsa‐miR‐766‐3p.

### 3.11 Expression of the marker genes in the validation set

**Figure 12:**
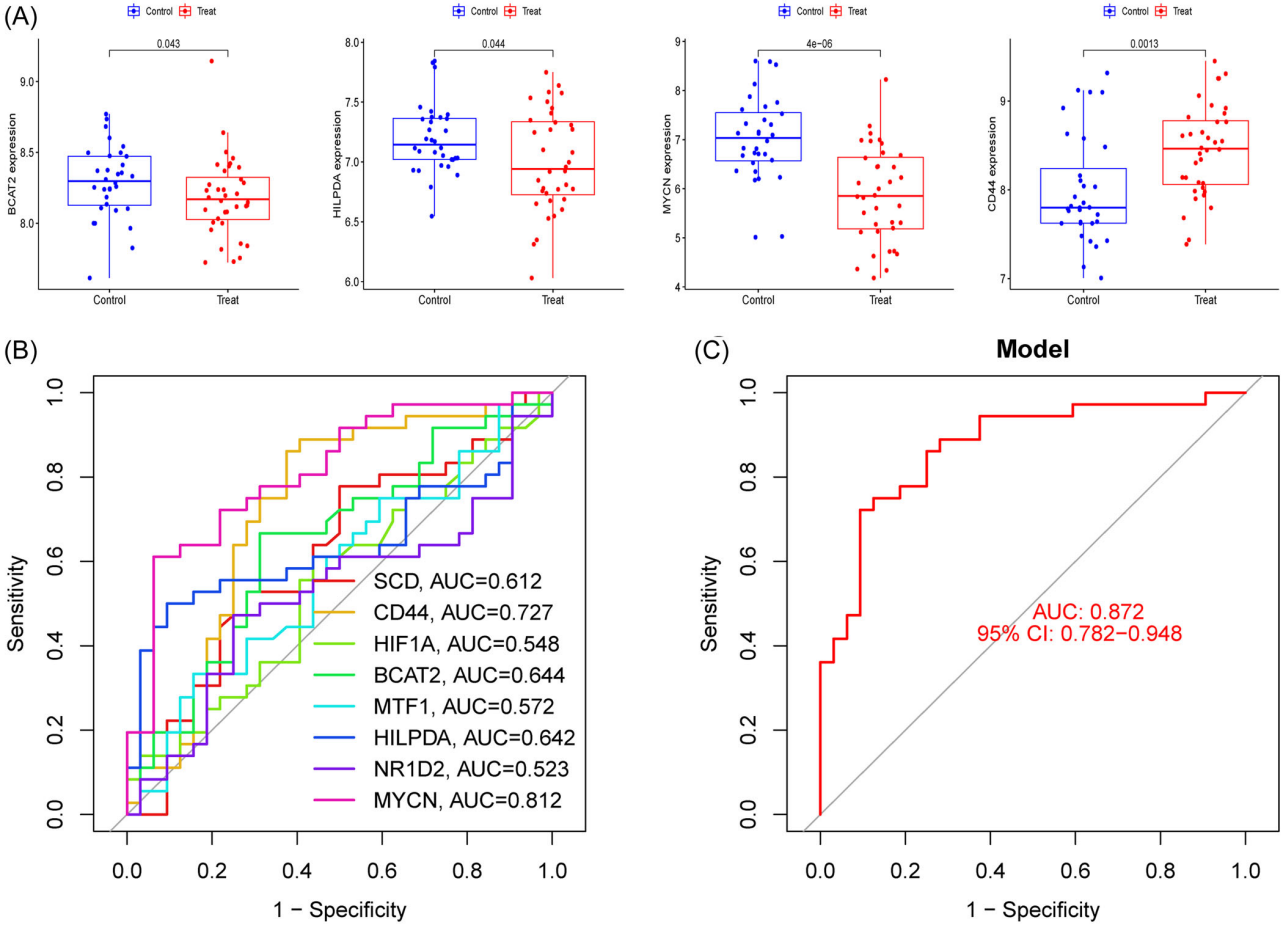
Expression status and diagnostic ability of marker genes in the validation set. (A) The expression of marker genes in the GSE76895 dataset. (B) ROC curves for the eight marker genes in the GSE76895 dataset. (C) Logistic regression model to determine the AUC of the validation set T2DM samples. AUC, area under the curve; ROC, receiver operating characteristic curve; T2DM, type 2 diabetes mellitus.

We verified the differential expression of these eight marker genes and the precision of the logistic regression model using the GSE76895 dataset. We also discovered that BCAT2, HILPDA, MYCN, and CD44 were differentially expressed, and the patterns of their expression were consistent with those in the GSE78721 dataset (Figure 12A). Among these, CD44 expression levels (*p* = .0013) were greater in T2DM patients than in controls, but BCAT2 expression levels (*p* = .043), HILPDA expression levels (*p* = .044), and MYCN expression levels (*p* = 4e‐06) were lower in T2DM patients. The ROC curves for the marker genes in the validation set are shown in Figure 12B. With an AUC of 0.872 (95% CI: 0.782–0.948), the logistic regression model based on eight marker genes continued to be extremely precise and specific in the validation group (Figure 12C).

### 3.12 Validation of CD44 and MYCN in T2DM and nondiabetic tissues

**Figure 13:**
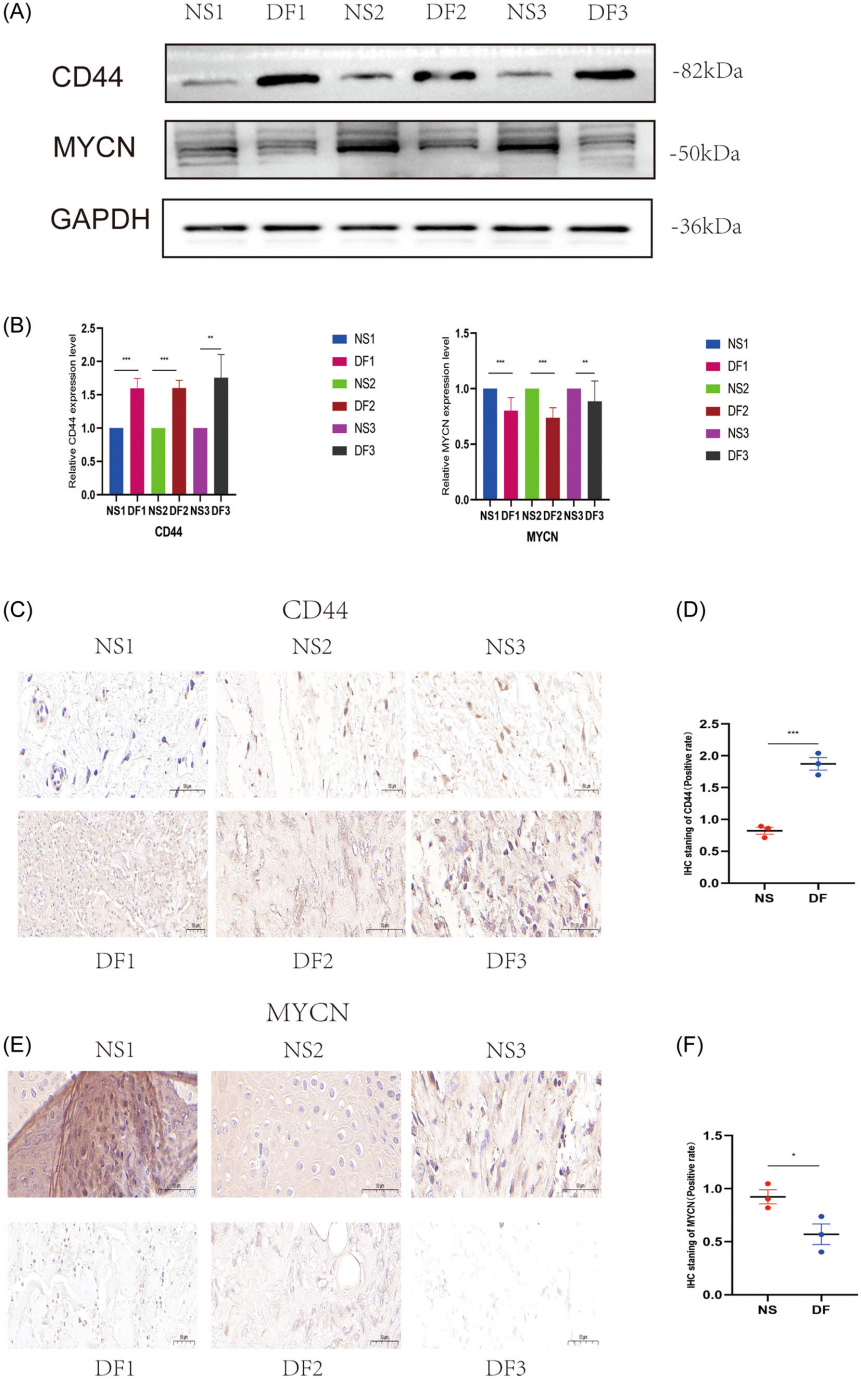
After ethical approval, we obtained ulcerated tissues from patients with diabetes and normal tissues from patients without diabetes and performed Western blot and immunohistochemical assays to detect the expression of CD44 and MYCN. (A, B) Protein blotting to detect CD44 and MYCN expression in ulcerated tissues from patients with diabetes and normal tissues from patients without diabetes. (C–F) IHC analysis and quantification of the percentage of CD44 versus MYCN in ulcerated tissues from patients with diabetes and normal tissues from patients without diabetes. Scale bar = 50 μm. *p < .05, **p < .01, ***p < .001. DF, diabetic foot; IHC, immunohistochemistry; NS, normal skin.

**Figure 14:**
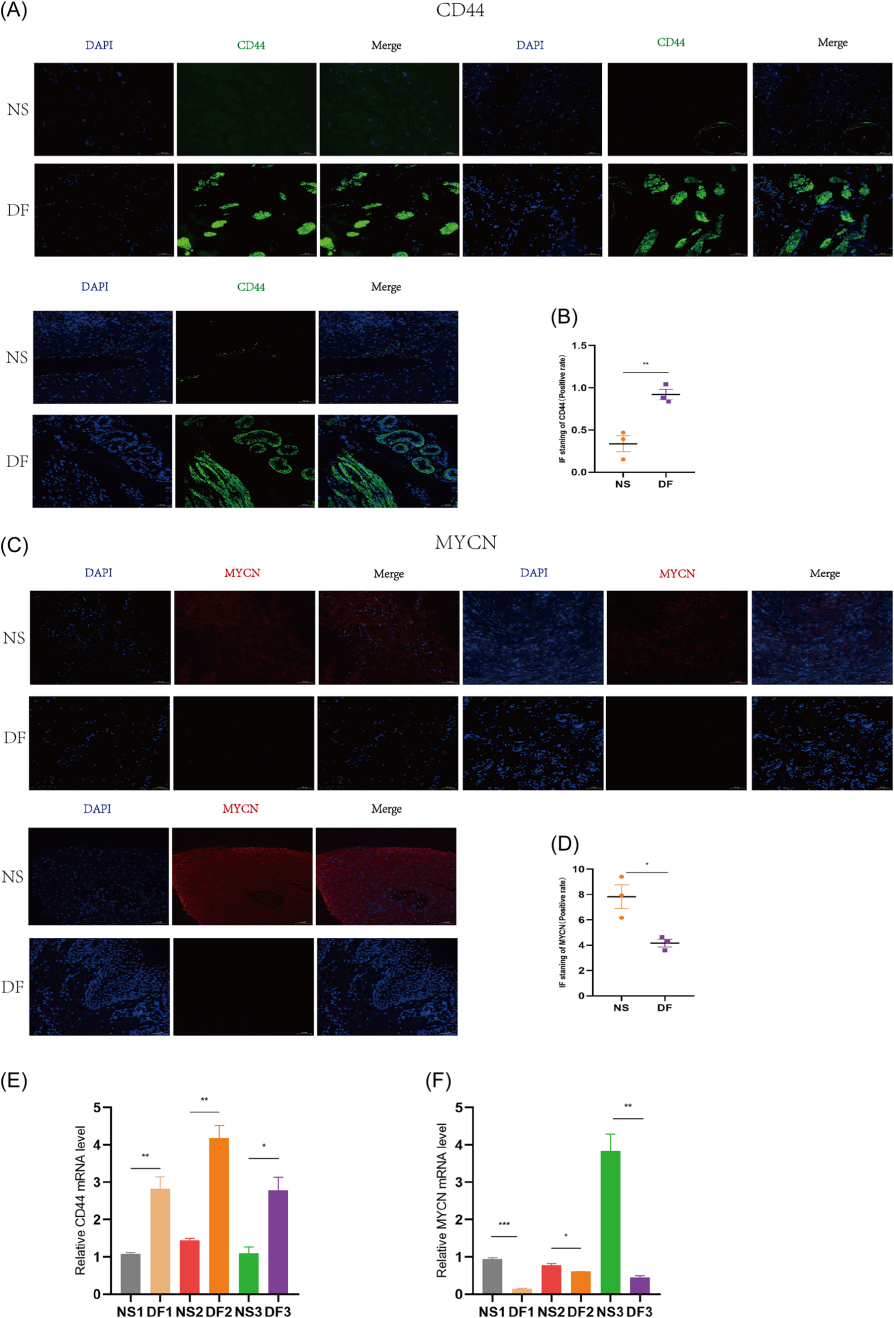
We further verified the expression data using immunofluorescence and qPCR. (A, B) Immunofluorescence assay and quantification of the percentage of CD44‐positive cells in diabetic ulcers and normal skin tissue. (C, D) Immunofluorescence assay and quantification of the percentage of MYCN‐positive cells in diabetic ulcers and normal skin tissue. Scale bar = 50 μm. (E, F) qRT‐PCR analysis of CD44 and MYCN mRNA levels is shown. *p < .05, **p < .01, ***p < .001. DF, diabetic foot; mRNA, messenger RNA; NS, normal skin.

We performed experimental validation by clinical tissues, and the experimental results were consistent with our bioinformatics analysis results. CD44 was indeed highly expressed in diabetic tissues and low in nondiabetic tissues; MYCN was low in diabetic tissues and high in nondiabetic tissues. As shown in Figure 13A,B, Western blotting showed a trend of high expression of CD44 and low expression of MYCN in diabetic tissues. In Figure 13C–F, IHC also showed a trend of high expression of CD44 and low expression of MYCN in diabetes tissues. We also performed IF validation, and the expression trends of CD44 in T2DM tissues were consistent with the Western blot and IHC results in Figure 14A,B. In IF, the expression trends of MYCN in diabetes tissues were consistent with the Western blot and IHC results (Figure 14C,D). Figure 14E,F show that qPCR was used to measure the expression levels of the biomarkers CD44 and MYCN. The expression levels of CD44 were likewise significantly high in diabetic tissues and low in nondiabetic tissues at the RNA level; MYCN expression levels were also low in diabetic tissues and high in nondiabetic tissues. We discovered that MYCN may have a protective effect against diabetes, and its overexpression may alleviate diabetes, reduce the occurrence of diabetes complications such as diabetic foot, and mitigate diabetes malignancy. CD44 has a destructive effect on diabetes, and knocking down its expression may help control the disease, reduce the likelihood of diabetic foot, and even reduce other diabetic complications, such as diabetic retinopathy and diabetic nephropathy. In the current study, we took tissues from patients with diabetic foot, which is one of the complications of diabetes, for the experimental group, and in future studies, we can take tissues from patients with other complications of diabetes, such as diabetic retinopathy and diabetic nephropathy, to verify again with ethical approval. Our research goals include in vitro experiments with MYCN overexpression, CD44 knockdown, or knockout in oxygen glucose deprivation‐induced diabetic cell models and in vivo experiments with MYCN overexpression, CD44 knockdown, or even knockout in high glucose‐ or advanced glycosylation end product (AGES)‐induced diabetic animal models in mice. Our findings have important implications for diabetes prevention and development, particularly for diabetic foot control. Furthermore, because diabetes is a heritable condition, studying MYCN and CD44 expression in parents and children with a family history of diabetes may offer new avenues for diabetes genetic research.

## 4 DISCUSSION

T2DM is an increasingly common metabolic disease and poses a significant public health burden.^28^, ^29^ In recent years, the diagnosis and treatment of T2DM have been increasingly studied. However, the prognosis for patients with T2DM remains poor due to the limited understanding of the pathogenesis of the disease and the numerous complications associated with drug therapy.^30^ Many studies now suggest that ferroptosis plays a substantial role in T2DM and its consequences and that it is a risk factor for T2DM development. However, the precise process remains unknown.^31^, ^32^ In this study, we screened eight DEGs associated with ferroptosis using two machine learning algorithms (LASSO and SVM) and finally constructed a diagnostic model based on these eight DEGs (SCD, CD44, HIF1A, BCAT2, MTF1, HILPDA, NR1D2, and MYCN). In the training cohort, the model had high predictive ability (AUC = 0.832). It also demonstrated great accuracy in the external validation cohort (AUC = 0.872), offering innovative information for the quick and early diagnosis of T2DM.

The most enriched GO categories, according to our analysis of KEGG pathway enrichment and GO enrichment, were responses to hypoxia, decreased oxygen levels, the RNA polymerase II transcription regulator complex, RNA polymerase II‐specific DNA‐binding transcription factor binding, and other processes. In addition, KEGG analysis indicated that these genes were also significantly enriched in the hypoxia inducible factor‐1 (HIF‐1) signaling pathway and ferroptosis. Erythropoietin is synthesized by HIFs.^33^ In diabetic nephropathy, HIF‐2 expression is decreased, while HIF‐1 expression is elevated. This has been demonstrated to be directly associated with the dysregulation of HIF signaling.^34^ In contrast, HIF‐1α inhibition and hypoxia‐mimicking HIF‐2α activation slow the progression of diabetic nephropathy.^35^, ^36^ In addition, hypoxia inhibits ferritin phagocytosis, increases mitochondrial ferritin, and protects against ferroptosis.^37^ Interestingly, the eight marker genes we screened included HIF1A and were highly expressed in T2DM. Among the thirty HIF1A target drugs retrieved, PX‐478, a small molecule inhibitor, preserved pancreatic β cell function and increased insulin levels in diabetic mice in the presence of high glucose metabolism overload.^38^ In addition, targeted knockdown of HIF1A in mice resulted in a reduction in the size of atherosclerotic lesions and a decrease in macrophage accumulation.^39^ Similar results were shown in PX‐478‐treated mice; there was a considerable decrease in the amount of atherosclerotic plaque in the aorta of these mice.^40^ Therefore, the HIF1A inhibitor PX‐478 has the potential to be used as a therapeutic agent against diabetes and its complications, but its exact mechanism remains elusive. HILPDA (hypoxia‐inducible lipid droplet‐associated protein) is another marker gene that we screened for an associated with hypoxia. HILPDA is a novel peroxisome proliferator‐activated receptor (PPAR) target that can be expressed in multiple tissues as a small lipid droplet‐associated protein.^41^ Different events, including hypoxia and beta‐adrenergic stimulation, and PPAR transcription factors increase the production of HILPDA.^41^, ^42^, ^43^, ^44^ HILPDA is regulated by PPARα through the upstream PPRE (PPAR response element), and targeted overexpression increases hepatic triglyceride (TG) storage by reducing TG secretion.^41^ Rodriguez et al.^44^ also showed a moderate reduction in TGs in the livers of mice with nonalcoholic steatohepatitis when they were specifically deficient in HILPDA. Norepinephrine (NE), a sympathetic neurotransmitter, increases extracellular fatty acid absorption and TG storage in macrophages by acting through HILPDA‐activated beta2‐adrenergic receptors (β2ARs) and decreasing free fatty acid release from TG‐loaded macrophages.^45^ Thus, HILPDA could also provide new therapeutic directions for metabolism‐related fatty liver disease as well as T2DM in the future. The results of our study show that hypoxia plays a very important role in the development of diabetes.

SCD is an enzyme that regulates lipids and helps to desaturate saturated fatty acids.^46^ The GSAV analysis of SCD also confirmed that it is involved in the biosynthesis of unsaturated fatty acids and the metabolism of fatty acids. Previous animal studies have shown that defects in SCD‐1 isoforms expressed in human tissues result in reduced lipid synthesis, increased lipid oxidation, enhanced insulin sensitivity, reduced hepatic glucose output and increased systemic glucose uptake.^47^, ^48^, ^49^ Rosiglitazone is an agent that targets SCD and is a thiazolidinedione that enhances insulin sensitivity. It was often used in the past for blood glucose control but is now used sparingly due to its significant cardiovascular side effects.^50^, ^51^ Animal studies have shown that SCD levels are elevated in obese rats but return to normal levels after rosiglitazone treatment.^52^ MK‐8245 is a potent liver‐targeted SCD inhibitor that lowers blood lipid levels and blood glucose levels and has been used in therapeutic trials to study T2DM.^53^, ^54^ Clofibrate and aramchol can lower blood lipid levels. They have been used to treat NAFLD and to indirectly delay the progression of T2DM.^55^, ^56^ However, the mechanisms underlying the association between SCD and T2DM need further investigation.

In addition, we verified the expression of two genes, CD44 and MYCN, in T2DM tissues and nondiabetic tissues by Western blotting, IHC staining, IF staining and qRT‐PCR, and the results were statistically significant. CD44 is a cell surface glycoprotein, and an increasing number of studies have indicated that CD44 is involved in the regulation of glucose metabolism.^57^ It has been shown that CD44 is elevated in diabetic tissues, which is also consistent with our analysis, and CD44 is correlated with insulin resistance and glycaemic control levels.^58^, ^59^, ^60^ Related studies have shown that hyaluronan (HA) activation of CD44 increases vulnerability of β cells to damage and increases insulin resistance, resulting in elevated blood glucose levels.^61^, ^62^ Conversely, disruption of the HA‐CD44 interaction reduces the inflammatory cascade involved in islet destruction and exerts an antidiabetic effect.^63^ In addition, treatment of obese mice with anti‐CD44 monoclonal antibody reduced fasting glucose levels, hepatic steatosis and insulin resistance to the level of treatment with metformin and pioglitazone.^64^ MYCN is a member of the MYC family of proto‐oncogenes and is associated with the development of many tumors, especially neuroblastoma (NB).^65^ In NB, MYCN maintains tumor growth by promoting fatty acid uptake.^66^ It has been shown that MYCN can increase glycolysis and is associated with nonobese DM.^67^, ^68^ However, studies related to MYCN and diabetes are still scarce.

The development of T2DM is closely related to disorders of immune status and function, and abnormal immune cell activation and the subsequent inflammatory environment make glycaemic control more difficult.^69^ In the present study, immune infiltration analysis revealed that activated DCs were highly expressed in T2DM. In contrast, resting DCs were expressed at low levels in T2DM. A study by Surendar et al.^70^ demonstrated that the activation state of myeloid DCs and plasmacytoid DCs in patients with diabetes may be caused by increased levels of granulocyte‐macrophage colony‐stimulating factor and other proinflammatory cytokines. Hyperinsulinaemia also stimulates DC activation and overexpression of the scavenger receptors SR‐A, CD36, and LOX‐1, which can boost DC oxidized low‐density lipoprotein absorption capacity.^71^, ^72^ Cardiovascular disease (CVD) remains the leading cause of death in T2DM patients.^73^ DCs play an important role in the development of CVD and atherosclerosis.^74^, ^75^ Recent research on relevant animals has also demonstrated that DCs concentrate primarily in perivascular adipose tissue (PVAT) and are linked to an excess of proinflammatory cytokines, which impairs the capacity of PVAT to increase vasorelaxation and perform anticontractile action in T2DM.^76^ For T2DM and its complications, immune infiltration can be thought of as a future therapeutic target.

The ferroptosis genes we looked for in T2DM were SCD, CD44, HIF1A, BCAT2, MTF1, HILPDA, NR1D2, and MYCN. We mainly discussed five genes (SCD, CD44, HIF1A, HILPDA, and MYCN) and selected two genes, CD44 and MYCN, for clinical validation. In conclusion, more research is needed to determine whether our projected noncoding RNAs and gene‐targeted medicines are involved in T2DM. Naturally, there are certain limitations to our study. First, we only experimentally verified the protein and RNA expression of CD44 and MYCN in diabetic and normal tissues, and more experiments are needed to further explore the mechanisms of these genes in T2DM. Second, a larger sample size of T2DM might increase the accuracy because the small sample size resulted in differential expression of several marker genes showing mistakes in external validation. Third, new FRGs are still to be found, and the FerrDb database is always being updated.

## 5 CONCLUSION

In this study, we identified eight hub genes (SCD, CD44, HIF1A, BCAT2, MTF1, HILPDA, NR1D2, and MYCN) that are closely associated with ferroptosis in T2DM. The three ferroptosis genes, HIF1A, HILPDA, and SCD, are strongly related to T2DM, hypoxia and lipid metabolism, providing new research directions for the development and treatment of T2DM and its complications. Based on these eight genes, we constructed a model with a high ability to diagnose T2DM. We also predicted the drugs corresponding to these eight genes and constructed a ceRNA network map. In addition, we verified the protein and RNA expression of CD44 and MYCN in diabetic and nondiabetic tissues by Western blotting, IHC, IF, and qRT‐PCR, and the results were statistically significant. The above findings suggest that further studies of ferroptosis may offer new therapeutic goals and biomarkers for patients with T2DM.

## AUTHOR CONTRIBUTIONS

**Sen Wang**: conceptualization; data curation; methodology; validation; writing—original draft. **Yongpan Lu**: data curation; formal analysis; validation; writing—original draft; writing—review & editing. **Tingting Chi**: data curation; validation. **Yixin Zhang**: data curation; writing—review & editing. **Yuli Zhao**: data curation. **Huimin Guo**: data curation. **Li Feng**: funding acquisition; resources; writing—review & editing.

## CONFLICT OF INTEREST STATEMENT

The authors declare no conflicts of interest.

## ETHICS STATEMENT

All experiments were approved by the ethics committee of the Affiliated Hospital of Shandong University of Traditional Chinese Medicine & Shandong Provincial Hospital of Traditional Chinese Medicine (Approval Number: AF/SC‐08/02.0). All procedures were carried out in strict accordance with the 1964 Declaration of Helsinki. All patients involved in this study provided informed consent before the study.

## Supporting information

Supplemental Figure 1. GSEA for 8 marker genes.

Supplemental Figure 2. Molecular formulae of 32 drugs from DrugBank database.

## Data Availability

The data that support the findings of this study are available from the corresponding author.
