# Supplementary figures and images for: Identification of ferroptosis‐related genes in type 2 diabetes mellitus based on machine learning
Source: Immun Inflamm Dis. 2023 Oct 11;11(10):e1036. doi: 10.1002/iid3.1036 (PMC10566453; doi:10.1002/iid3.1036)

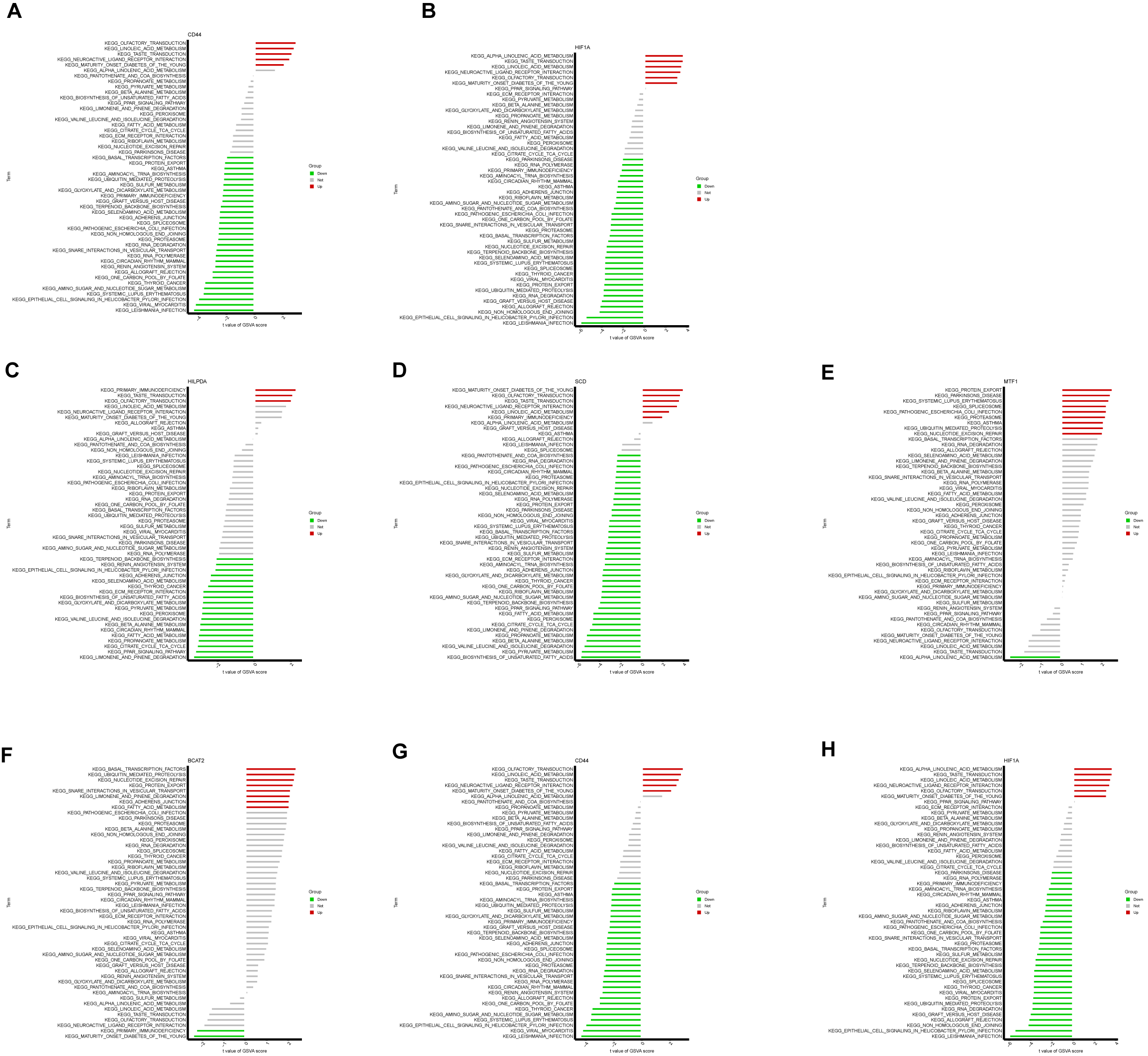

Supplement: Supplementary file 1 — Supplemental Figure 1. GSEA for 8 marker genes. [file IID3-11-e1036-s001.tif]

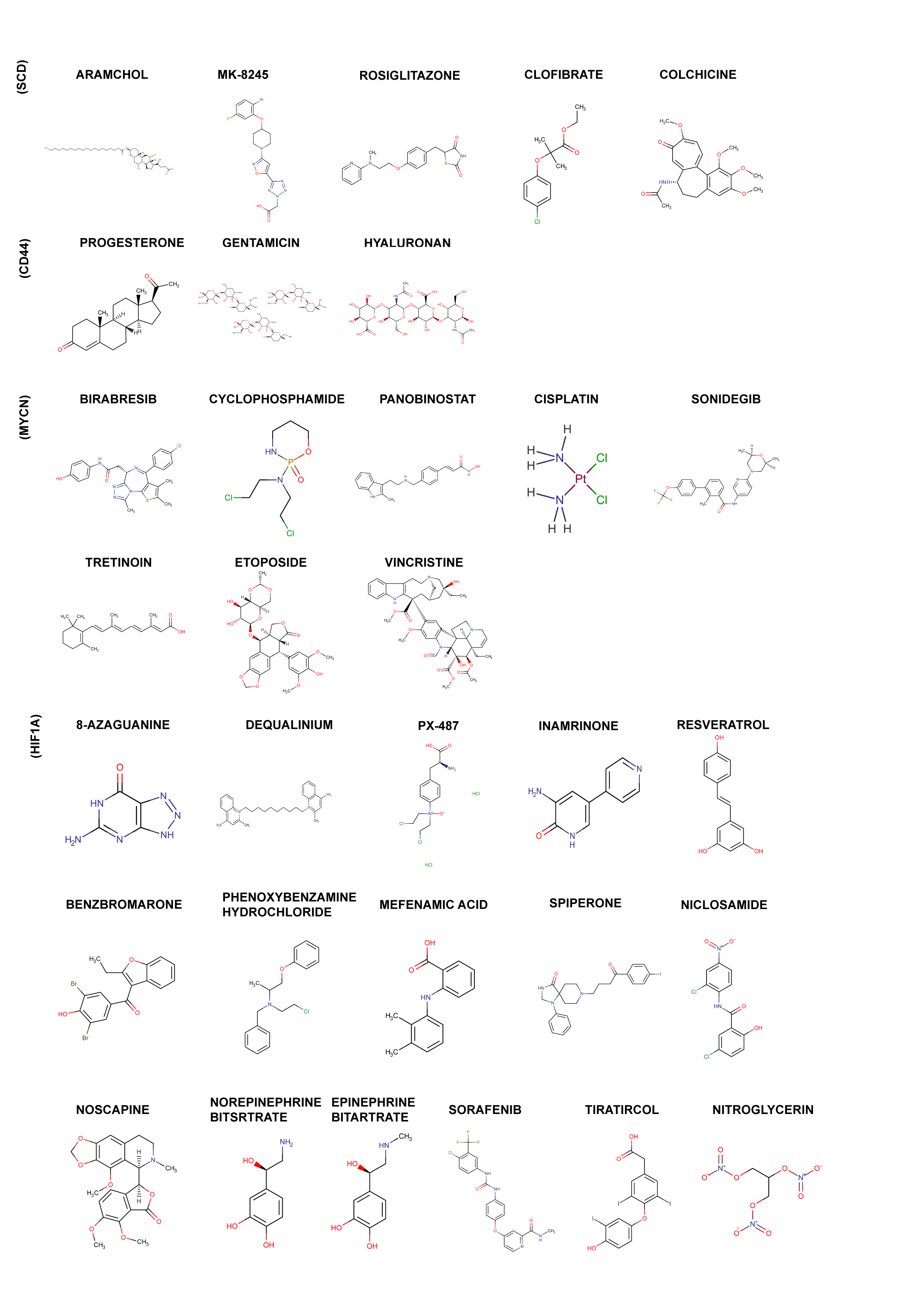

Supplement: Supplementary file 2 — Supplemental Figure 2. Molecular formulae of 32 drugs from DrugBank database. [file IID3-11-e1036-s002.tif]
